# Feature Selection and Classification of MAQC-II Breast Cancer and Multiple Myeloma Microarray Gene Expression Data

**DOI:** 10.1371/journal.pone.0008250

**Published:** 2009-12-11

**Authors:** Qingzhong Liu, Andrew H. Sung, Zhongxue Chen, Jianzhong Liu, Xudong Huang, Youping Deng

**Affiliations:** 1 Department of Computer Science and Institute for Complex Additive Systems Analysis, New Mexico Tech, Socorro, New Mexico, United States of America; 2 Department of Epidemiology & Biostatistics, Robert Stempel College of Public Health & Social Work, Florida International University, Miami, Florida, United States of America; 3 QuantumBio Inc., State College, Pennsylvania, United States of America; 4 Division of Nuclear Medicine and Molecular Imaging and Center for Advanced Medical Imaging, Department of Radiology, Brigham and Women's Hospital and Harvard Medical School, Boston, Massachusetts, United States of America; 5 SpecPro, Vicksburg, Mississippi, United States of America; 6 Department of Biology Science, the University of Southern Mississippi, Hattiesburg, Mississippi, United States of America; Memorial Sloan Kettering Cancer Center, United States of America

## Abstract

Microarray data has a high dimension of variables but available datasets usually have only a small number of samples, thereby making the study of such datasets interesting and challenging. In the task of analyzing microarray data for the purpose of, e.g., predicting gene-disease association, feature selection is very important because it provides a way to handle the high dimensionality by exploiting information redundancy induced by associations among genetic markers. Judicious feature selection in microarray data analysis can result in significant reduction of cost while maintaining or improving the classification or prediction accuracy of learning machines that are employed to sort out the datasets. In this paper, we propose a gene selection method called Recursive Feature Addition (RFA), which combines supervised learning and statistical similarity measures. We compare our method with the following gene selection methods:

- Support Vector Machine Recursive Feature Elimination (SVMRFE)
- Leave-One-Out Calculation Sequential Forward Selection (LOOCSFS)
- Gradient based Leave-one-out Gene Selection (GLGS)

To evaluate the performance of these gene selection methods, we employ several popular learning classifiers on the MicroArray Quality Control phase II on predictive modeling (MAQC-II) breast cancer dataset and the MAQC-II multiple myeloma dataset. Experimental results show that gene selection is strictly paired with learning classifier. Overall, our approach outperforms other compared methods. The biological functional analysis based on the MAQC-II breast cancer dataset convinced us to apply our method for phenotype prediction. Additionally, learning classifiers also play important roles in the classification of microarray data and our experimental results indicate that the Nearest Mean Scale Classifier (NMSC) is a good choice due to its prediction reliability and its stability across the three performance measurements: Testing accuracy, MCC values, and AUC errors.

## Introduction

Using microarray techniques, researchers can measure the expression levels for tens of thousands of genes in a single experiment. This ability allows scientists to investigate the functional relationship between the cellular and physiological processes of biological organisms and genes at a genome-wide level. The preprocessing procedure for the raw microarray data consists of background correction, normalization, and summarization. After preprocessing, a high level analysis, such as gene selection, classification, or clustering, is applied to profile the gene expression patterns [1]. In the high-level analysis, partitioning genes into closely related groups across time and classifying patients into different health statuses based on selected gene signatures have become two main tracks of microarray data analysis in the past decade [2]–[6]. Various standards related to systems biology are discussed by Brazma *et al.* [7]. When sample sizes are substantially smaller than the number of features or genes, statistical modeling and inference issues become challenging as the familiar “large p small n problem” arises. Designing feature selection methods that lead to reliable and accurate predictions by learning classifiers, therefore, is an issue of great theoretical as well as practical importance in high dimensional data analysis.

To address the “curse of dimensionality” problem, three basic strategies have been proposed for feature selection: filtering, wrapper, and embedded methods. Filtering methods select subset features independently from the learning classifiers and do not incorporate learning [8]–[11]. One of the weaknesses of filtering methods is that they only consider the individual feature in isolation and ignore the possible interaction among features. Yet the combination of certain features may have a net effect that does not necessarily follow from the individual performance of features in that group [12]. A consequence of the filtering methods is that we may end up with selecting groups of highly correlated features/genes, which present redundant information to the learning classifier to ultimately worsen its performance. Also, if there is a practical limit on the number of features to be chosen, one may not be able to include all informative features.

To avoid the weakness of filtering methods, wrapper methods wrap around a particular learning algorithm that can assess the selected feature subsets in terms of the estimated classification errors and then build the final classifier [13]. Wrapper methods use a learning machine to measure the quality of subsets of features. One recent well-known wrapper method for feature/gene selection is Support Vector Machine Recursive Feature Elimination (SVMRFE) [14], which refines the optimum feature set by using Support Vector Machines (SVM). The idea of SVMRFE is that the orientation of the separating hyper-plane found by the SVM can be used to select informative features; if the plane is orthogonal to a particular feature dimension, then that feature is informative, and vice versa. In addition to microarray data analysis, SVMRFE has been widely used in high-throughput biological data analyses and other areas involving feature selection and pattern classification [15].

Wrapper methods can noticeably reduce the number of features and significantly improve the classification accuracy [16], [17]. However, wrapper methods have the drawback of high computational load, making them less desirable as the dimensionality increases. The embedded methods perform feature selection simultaneously with learning classifiers to achieve better computational efficiency than wrapper methods while maintaining similar performance. LASSO [18], [19], logic regression with the regularized Laplacian prior [20], and Bayesian regularized neural network with automatic relevance determination [21] are examples of embedded methods.

To improve classification of microarray data, Zhou and Mao proposed SFS-LS bound and SFFS-LS bound algorithms for optimal gene selection by combining the sequential forward selection (SFS) and sequential floating forward selection (SFFS) with LS (Least Squares) bound measure [22]. Tang *et al.* designed two methods of gene selection, leave-one-out calculation sequential forward selection (LOOCSFS) and the gradient based leave-one-out gene selection (GLGS) [23]. Diaz-Uriarte and De Andres [24] presented a method for gene selection by calculating the out of bag errors with random forest [25].

In human genetic research, exploiting information redundancy from highly correlated genes can potentially reduce the cost and simultaneously improve the reliability and accuracy of learning classifiers that are employed in data analysis. To exploit the information redundancy that exists among the huge number of variables and improve classification accuracy of microarray data, we propose a gene selection method, Recursive Feature Addition (RFA), which is based on supervised learning and similarity measures. We compare RFA with SVMRFE, LOOCSFS, and GLGS by using the MAQC-II breast cancer dataset to predict pre-operative treatment response (pCR) and estrogen receptor status (erpos) and compare RFA with SVMRFE and LOOCSFS on the MAQC-II multiple myeloma dataset to predict the overall survival milestone outcome (OSMO, 730-day cutoff) and to predict event-free survival milestone outcome (EFSMO, 730-day cutoff).

## Results

### Results on MAQC-II Breast Cancer Dataset

We compare MSC-based RFA methods with GLGS, LOOCSFS, and SVMRFE on MAQC-II breast cancer dataset. Tables 1 to [Table pone-0008250-t002] [Table pone-0008250-t003] [Table pone-0008250-t004] [Table pone-0008250-t005] [Table pone-0008250-t006] [Table pone-0008250-t007] [Table pone-0008250-t008] [Table pone-0008250-t009] 10 list the cancer related genes of the first 100 features selected by these methods. In comparison to GLGS, LOOCSFS, and SVMRFE, RFA is associated with a greater number of currently known cancer related genes for prediction of pCR and a smaller number of currently known cancer related genes for prediction of erpos. Since disease status is not simply related to the number of these cancer related genes, we obtain the prediction performance by running multiple experiments, and compare the average prediction performance using the following measurements: testing accuracy, Matthews Correlation Coefficients (MCC) [26], [27] that has been used in MAQC-II consortium [28], and area under the receiver operating curve (AUC) errors with classifiers NMSC, NBC, SVM and UDC (uncorrelated normal based quadratic Bayes classifier) [29].

**Table 1:** The 34 cancer related genes of the 100 features selected by NBC-MSC on original training group of MAQC-II breast cancer data for pCR prediction.

Symbol	Synonym(s)	Entrez Gene Name	Affymetrix
ALB	ALB1, ALBUMIN, Albumin 1, Albza, DKFZp779N1935, PRO0883, PRO0903, PRO1341, SA, SERUM ALBUMIN, SERUM ALBUMIN CHAIN A, Serum albumin precursor	albumin	214837_at
C10ORF81	9930023K05RIK, bA211N11.2, FLJ23537, HEL185, MGC99964, RGD1559884, RP11-211N11.2	chromosome 10 open reading frame 81	219857_at
CDK10	BC017131, MGC112847, PISSLRE	cyclin-dependent kinase 10	210622_x_at
CEACAM1	bb-1, BGP, BGP1, BGPA, Bgpd, BGPI, BGPR, C-CAM, C-CAM1, CCAM105, CD66, CD66A, Cea-1, Cea-7, CEACAM1-4L, ECTO ATPASE, HV2, mCEA1, Mhv-1, MHVR, MHVR1, mmCGM1, mmCGM1a, mmCGM2, Pp120	carcinoembryonic antigen-related cell adhesion molecule 1 (biliary glycoprotein)	211889_x_at
CHRNB4	Acrb-4, NACHR BETA4	cholinergic receptor, nicotinic, beta 4	207516_at
CR1	C3b/C4b receptor, C3BR, CD35, CD46, Cr1l, Crry, KN, Mcp, mCRY, MGC102484, SCR1	complement component (3b/4b) receptor 1 (Knops blood group)	217552_x_at
CXCL3	Cinc-2, CINC-2a, CINC-2b, Cinc3, Cxcl2, Dcip1, Gm1960, GRO ALPHA, GRO BETA, GRO GAMMA, GRO1, Gro2, GRO3, GROA, GROb, GROg, KC, MGSA, Mgsa-b, MIP-2, MIP-2a, MIP-2b, Mip2 alpha, N51, Scyb, Scyb2, SCYB3	chemokine (C-X-C motif) ligand 3	207850_at
CXCL13	ANGIE, ANGIE2, BCA-1, BLC, BLC1, BLR1L, CXC CHEMOKINE, Loc498335, SCYB13	chemokine (C-X-C motif) ligand 13	205242_at
DKK1	Dkk1 predicted, mdkk-1, SK	dickkopf homolog 1 (Xenopus laevis)	204602_at
DRD2	D2, D2 DOPAMINE RECEPTOR, D2a dopamine receptor, D2DR, D2R, D2S, DOPAMINE D2 RECEPTOR, Dr2	dopamine receptor D2	216924_s_at
EED	HEED, l(7)5Rn, l7Rn5, lusk, WAIT-1	embryonic ectoderm development	209572_s_at
GINS3	2700085M18Rik, AI616142, FLJ13912, PSF3, RGD1308153	GINS complex subunit 3 (Psf3 homolog)	218719_s_at
GPS2	AI505953, AMF-1, MGC104294, MGC119287, MGC119288, MGC119289	G protein pathway suppressor 2	209350_s_at
GRIA2	GLUR-B, GluR-K2, GLUR2, GLUR2 IONOTROPIC, HBGR2	glutamate receptor, ionotropic, AMPA 2	205358_at
GSN	DKFZp313L0718, GELSOLIN, MGC28083, MGC95032	gelsolin (amyloidosis, Finnish type)	214040_s_at
IFNAR1	ALPHA CHAIN OF TYPE I IFNR, AVP, BETA R1, CD118, Ifar, IFN RECEPTOR TYPE 1, IFN TYPE 1 RECEPTOR, IFN-alpha-beta-R, IFN-ALPHA-REC, IFNalpha/betaR, IFNAR, IFNBR, IFRC, Infar, INFAR1, Interferon Receptor, LOC284829, Type I infr	interferon (alpha, beta and omega) receptor 1	204191_at
ITGB4	AA407042, C230078O20, CD104, INTEGRIN-BETA 4	integrin, beta 4	204989_s_at
IVD	1300016K07Rik, 6720455E18Rik, ACAD2, AI463340, Isovaleryl-Coa Dehydrogenase	isovaleryl Coenzyme A dehydrogenase	216958_s_at
KL	ALPHA KLOTHO, alpha-kl, KLOTHO	klotho	205978_at
N4BP1	AI481586, C81621, FLJ31821, KIAA0615, MGC176730, MGC7607, RGD1305179	NEDD4 binding protein 1	32069_at
NAIP	AV364616, BIRC1, BIRC1A, Birc1b, Birc1e, BIRC1F, D13Lsd1, FLJ18088, FLJ42520, FLJ58811, LGN1, LOC652755, Naip-rs1, Naip-rs3, Naip-rs4, Naip-rs4A, Naip1, Naip2, Naip5, Naip6, NLRB1, psiNAIP, RGD1559914	NLR family, apoptosis inhibitory protein	204861_s_at
NDST1	1200015G06RIK, HSNST, HSST, HSST1, NST1	N-deacetylase/N-sulfotransferase (heparan glucosaminyl) 1	202608_s_at
PAICS	2610511I09Rik, ADE2, ADE2H1, AIRC, DKFZp781N1372, MGC1343, MGC5024, MGC93240, PAIS	phosphoribosylaminoimidazole carboxylase, phosphoribosylaminoimidazole succinocarboxamide synthetase	214664_at
PAWR	PAR-4	PRKC, apoptosis, WT1, regulator	204005_s_at
PDPK1	MGC20087, MGC35290, PDK1, PRO0461	3-phosphoinositide dependent protein kinase-1	204524_at
PHLDA1	DT1P1B11, MGC131738, PHRIP, PQ-RICH, Proline- and glutamine-rich, TDAG, TDAG51	pleckstrin homology-like domain, family A, member 1	218000_s_at
PPP1R15A (includes EG:23645)	9630030H21, GADD34, MYD116, Myeloid Differentiation, Peg-3, PP1 REGULATORY SUBUNIT, Ppp1r15a	protein phosphatase 1, regulatory (inhibitor) subunit 15A	202014_at
RASSF1	123F2, AA536941, AU044980, D4Mgi37, MGC94319, NORE2A, PTS, Rassf1A, Rassf1B, Rassf1C, RDA32, REH3P21	Ras association (RalGDS/AF-6) domain family member 1	204346_s_at
SYT1	AW124717, DKFZp781D2042, G630098F17Rik, P65, SVP65, SYNAPTOTAGMIN 1, SYT	synaptotagmin I	203999_at
TACSTD2	C80403, EGP-1, GA733, GA733-1, Ly97, M1S1, MGC141612, MGC141613, MGC72570, Prp1, TROP2	tumor-associated calcium signal transducer 2	202286_s_at
TEK	AA517024, CD202B, Hyk, MGC139569, TIE-2, VMCM, VMCM1	TEK tyrosine kinase, endothelial	206702_at
TTF1	AV245725, RGD1565673, Ttf-I	transcription termination factor, RNA polymerase I	204772_s_at
ZEB1	3110032K11Rik, AREB6, BZP, DELTA-EF1, MEB1, MGC133261, NIL-2-A, Nil2, Tcf18, TCF8, TCP8, TF8, TRANSCRIPTION FACTOR 8, ZEB, ZFHEP, Zfhep2, ZFHX1A, Zfx1a, Zfx1ha, [delta]EF1	zinc finger E-box binding homeobox 1	212758_s_at
ZNF10	KOX1	zinc finger protein 10	216350_s_at

**Table 2 pone-0008250-t002:** The 34 cancer related genes of the 100 features selected by NMSC-MSC on original training group of MAQC-II breast cancer data for pCR prediction.

Symbol	Synonym(s)	Entrez Gene Name	Affymetrix
ALB	ALB1, ALBUMIN, Albumin 1, Albza, DKFZp779N1935, PRO0883, PRO0903, PRO1341, SA, SERUM ALBUMIN, SERUM ALBUMIN CHAIN A, Serum albumin precursor	albumin	214837_at
ARAF	1200013E08Rik, ARAF1, AW495444, PKS, PKS2, RAFA1	v-raf murine sarcoma 3611 viral oncogene homolog	201895_at
C10ORF81	9930023K05RIK, bA211N11.2, FLJ23537, HEL185, MGC99964, RGD1559884, RP11-211N11.2	chromosome 10 open reading frame 81	219857_at
CDH1	AA960649, Arc-1, Cadherin 1, CD324, CDHE, CSEIL, E-CADHERIN, E-CADHERIN 120 KDA, ECAD, L-CAM, MGC107495, Um, UVO, uvomorulin	cadherin 1, type 1, E-cadherin (epithelial)	201131_s_at
CDK10	BC017131, MGC112847, PISSLRE	cyclin-dependent kinase 10	210622_x_at
CEACAM1	bb-1, BGP, BGP1, BGPA, Bgpd, BGPI, BGPR, C-CAM, C-CAM1, CCAM105, CD66, CD66A, Cea-1, Cea-7, CEACAM1-4L, ECTO ATPASE, HV2, mCEA1, Mhv-1, MHVR, MHVR1, mmCGM1, mmCGM1a, mmCGM2, Pp120	carcinoembryonic antigen-related cell adhesion molecule 1 (biliary glycoprotein)	211889_x_at
CEBPE	C/EBP EPSILON, C/EBPe, C/EPBe, CRP1, Gm294, MGC124002, MGC124003	CCAAT/enhancer binding protein (C/EBP), epsilon	214523_at
CHRNB4	Acrb-4, NACHR BETA4	cholinergic receptor, nicotinic, beta 4	207516_at
CR1	C3b/C4b receptor, C3BR, CD35, CD46, Cr1l, Crry, KN, Mcp, mCRY, MGC102484, SCR1	complement component (3b/4b) receptor 1 (Knops blood group)	217552_x_at
CXCL3	Cinc-2, CINC-2a, CINC-2b, Cinc3, Cxcl2, Dcip1, Gm1960, GRO ALPHA, GRO BETA, GRO GAMMA, GRO1, Gro2, GRO3, GROA, GROb, GROg, KC, MGSA, Mgsa-b, MIP-2, MIP-2a, MIP-2b, Mip2 alpha, N51, Scyb, Scyb2, SCYB3	chemokine (C-X-C motif) ligand 3	207850_at
CXCL13	ANGIE, ANGIE2, BCA-1, BLC, BLC1, BLR1L, CXC CHEMOKINE, Loc498335, SCYB13	chemokine (C-X-C motif) ligand 13	205242_at
DRD2	D2, D2 DOPAMINE RECEPTOR, D2a dopamine receptor, D2DR, D2R, D2S, DOPAMINE D2 RECEPTOR, Dr2	dopamine receptor D2	216924_s_at
DYRK1A	2310043O08Rik, D16Ertd272e, D16Ertd493e, DUAL-SPECIFICITY TYROSINE-(Y)-PHOSPHORYLATION REGULATED KINASE 1A, DYRK, DYRK1, HP86, MGC150253, MGC150254, mmb, MNB, MNBH, Mp86, PSK47	dual-specificity tyrosine-(Y)-phosphorylation regulated kinase 1A	211541_s_at
EPOR	EP-R, ERYTHROPOIETIN RECEPTOR, MGC108723, MGC138358	erythropoietin receptor	215054_at
FAM153A	KIAA0752, NY-REN-7	family with sequence similarity 153, member A	211166_at
GINS3	2700085M18Rik, AI616142, FLJ13912, PSF3, RGD1308153	GINS complex subunit 3 (Psf3 homolog)	218719_s_at
GRIA2	GLUR-B, GluR-K2, GLUR2, GLUR2 IONOTROPIC, HBGR2	glutamate receptor, ionotropic, AMPA 2	205358_at
GSN	DKFZp313L0718, GELSOLIN, MGC28083, MGC95032	gelsolin (amyloidosis, Finnish type)	214040_s_at
IFNAR1	ALPHA CHAIN OF TYPE I IFNR, AVP, BETA R1, CD118, Ifar, IFN RECEPTOR TYPE 1, IFN TYPE 1 RECEPTOR, IFN-alpha-beta-R, IFN-ALPHA-REC, IFNalpha/betaR, IFNAR, IFNBR, IFRC, Infar, INFAR1, Interferon Receptor, LOC284829, Type I infr	interferon (alpha, beta and omega) receptor 1	204191_at
ITGB4	AA407042, C230078O20, CD104, INTEGRIN-BETA 4	integrin, beta 4	204989_s_at
KL	ALPHA KLOTHO, alpha-kl, KLOTHO	klotho	205978_at
LPAR1	5031439C20, AI326300, clone 4.9, EDG2, ENDOTHELIAL DIFFERENTIATION LYSOPHOSPHATIDIC ACID G-PROTEIN-COUPLED RECEPTOR 2, Gpcr26, GPR26, Kdt2, LPA receptor 1, LPA1, LPA1 RECEPTOR, LPA2, LYSOPHOSPHATIDIC ACID G-PROTEIN-COUPLED RECEPTOR, MGC105279, MGC29102, Mrec1.3, rec.1.3, vzg-1	lysophosphatidic acid receptor 1	204037_at
MCF2	B230117G22Rik, DBL, MGC159138, RGD1566098	MCF.2 cell line derived transforming sequence	208017_s_at
MYO10	AW048724, D15Ertd600e, FLJ10639, FLJ21066, FLJ22268, FLJ43256, KIAA0799, MGC131988, mKIAA0799, Myo10 (predicted), myosin-X	myosin X	201976_s_at
NAIP	AV364616, BIRC1, BIRC1A, Birc1b, Birc1e, BIRC1F, D13Lsd1, FLJ18088, FLJ42520, FLJ58811, LGN1, LOC652755, Naip-rs1, Naip-rs3, Naip-rs4, Naip-rs4A, Naip1, Naip2, Naip5, Naip6, NLRB1, psiNAIP, RGD1559914	NLR family, apoptosis inhibitory protein	204860_s_at
NDST1	1200015G06RIK, HSNST, HSST, HSST1, NST1	N-deacetylase/N-sulfotransferase (heparan glucosaminyl) 1	202608_s_at
PHLDA1	DT1P1B11, MGC131738, PHRIP, PQ-RICH, Proline- and glutamine-rich, TDAG, TDAG51	pleckstrin homology-like domain, family A, member 1	218000_s_at
PPP1R15A (includes EG:23645)	9630030H21, GADD34, MYD116, Myeloid Differentiation, Peg-3, PP1 REGULATORY SUBUNIT, Ppp1r15a	protein phosphatase 1, regulatory (inhibitor) subunit 15A	202014_at
RASSF1	123F2, AA536941, AU044980, D4Mgi37, MGC94319, NORE2A, PTS, Rassf1A, Rassf1B, Rassf1C, RDA32, REH3P21	Ras association (RalGDS/AF-6) domain family member 1	204346_s_at
SIAH1	AA982064, AI853500, D9MGI7, FLJ08065, hSIAH1, HUMSIAH, SIAH, Siah1a, Sinh1a	seven in absentia homolog 1 (Drosophila)	202981_x_at
TTF1	AV245725, RGD1565673, Ttf-I	transcription termination factor, RNA polymerase I	204772_s_at
VAMP2	FLJ11460, mVam2, RATVAMPB, RATVAMPIR, SYB, SYB2, SYNAPTOBREVIN 2, Vamp ii	vesicle-associated membrane protein 2 (synaptobrevin 2)	201557_at
ZNF10	KOX1	zinc finger protein 10	216350_s_at
ZNF205	4933429B21, AI835008, Krox-8, Zfp13, ZINC FINGER PROTEIN 205, ZNF210	zinc finger protein 205	206416_at

**Table 3 pone-0008250-t003:** The 26 cancer related genes of the 100 features selected by GLGS on original training group of MAQC-II breast cancer data for pCR prediction.

Symbol	Synonym(s)	Entrez Gene Name	Affymetrix
ADAM15	MDC15, METARGIDIN, tMDCVI	ADAM metallopeptidase domain 15	217007_s_at
ARAF	1200013E08Rik, ARAF1, AW495444, PKS, PKS2, RAFA1	v-raf murine sarcoma 3611 viral oncogene homolog	201895_at
ASNS	AS, ASPARAGINE SYNTHETASE, MGC93148, TS11	asparagine synthetase	205047_s_at
B4GALT5	9430078I07Rik, AW049941, AW539721, BETA4-GALT-IV, beta4Gal-T5, beta4GalT-V, gt-V, MGC138470	UDP-Gal:betaGlcNAc beta 1,4- galactosyltransferase, polypeptide 5	221485_at
CEACAM1	bb-1, BGP, BGP1, BGPA, Bgpd, BGPI, BGPR, C-CAM, C-CAM1, CCAM105, CD66, CD66A, Cea-1, Cea-7, CEACAM1-4L, ECTO ATPASE, HV2, mCEA1, Mhv-1, MHVR, MHVR1, mmCGM1, mmCGM1a, mmCGM2, Pp120	carcinoembryonic antigen-related cell adhesion molecule 1 (biliary glycoprotein)	211889_x_at
CXCL3	Cinc-2, CINC-2a, CINC-2b, Cinc3, Cxcl2, Dcip1, Gm1960, GRO ALPHA, GRO BETA, GRO GAMMA, GRO1, Gro2, GRO3, GROA, GROb, GROg, KC, MGSA, Mgsa-b, MIP-2, MIP-2a, MIP-2b, Mip2 alpha, N51, Scyb, Scyb2, SCYB3	chemokine (C-X-C motif) ligand 3	207850_at
CYCS (includes EG:54205)	CYC, CYCS, CYCSA, CYCT, CYCTA, CYTC, CYTOCHROME C, HCS, MGC93634, T-Cc	cytochrome c, somatic	208905_at
DRD2	D2, D2 DOPAMINE RECEPTOR, D2a dopamine receptor, D2DR, D2R, D2S, DOPAMINE D2 RECEPTOR, Dr2	dopamine receptor D2	216924_s_at
EGFR	9030024J15RIK, AI552599, EGF RECEPTOR, EGF-TK, EGFR1, EGFRec, ER2, ERBB, ERBB1, Errp, HER1, MENA, PIG61, wa-2, Wa5	epidermal growth factor receptor (erythroblastic leukemia viral (v-erb-b) oncogene homolog, avian)	201983_s_at
FBLN1	FBLN, FIBULIN 1	fibulin 1	207834_at
FIS1	2010003O14Rik, CGI-135, Riken cDNA 2010003o14, TTC11	fission 1 (mitochondrial outer membrane) homolog (S. cerevisiae)	218034_at
FOXC1	ARA, ch, fkh-1, FKHL7, FLJ11796, FLJ11796 FIS, FREAC-3, frkhda, IGDA, IHG1, IRID1, Mf1, Mf4, rCG 44068, rCG_44068	forkhead box C1	213260_at
FTL	FERRITIN LIGHT CHAIN, FTL1, Ftl2, L-FERRITIN, MGC102130, MGC102131, MGC118079, MGC118080, MGC71996, RGD1560687, RGD1561055, RGD1566189, YB24D08	ferritin, light polypeptide	201265_at
GFRA1	AU042498, GDNFR, Gdnfr alpha, GDNFRA, GFR-ALPHA-1, GRFA1, MGC23045, Ret, RET1L, RETL1, TRNR1	GDNF family receptor alpha 1	205696_s_at
ITGB4	AA407042, C230078O20, CD104, INTEGRIN-BETA 4	integrin, beta 4	204989_s_at
KL	ALPHA KLOTHO, alpha-kl, KLOTHO	klotho	205978_at
LEF1	3000002B05, AI451430, DKFZp586H0919, LEF, TCF/LEF, TCF1ALPHA	lymphoid enhancer-binding factor 1	221557_s_at
LMO4	A730077C12Rik, Crp3, Etohi4, MGC105593	LIM domain only 4	209205_s_at
LPAR2	EDG-4, FLJ93869, IPA2, LPA receptor 2, LPA2, LPA2 RECEPTOR, Pbx4, RGD1561336	lysophosphatidic acid receptor 2	206723_s_at
MKI67	D630048A14Rik, KI-67, KIA, MIB1, MIKI67A, MKI67A	antigen identified by monoclonal antibody Ki-67	212022_s_at
NAIP	AV364616, BIRC1, BIRC1A, Birc1b, Birc1e, BIRC1F, D13Lsd1, FLJ18088, FLJ42520, FLJ58811, LGN1, LOC652755, Naip-rs1, Naip-rs3, Naip-rs4, Naip-rs4A, Naip1, Naip2, Naip5, Naip6, NLRB1, psiNAIP, RGD1559914	NLR family, apoptosis inhibitory protein	204861_s_at
PPP1R15A (includes EG:23645)	9630030H21, GADD34, MYD116, Myeloid Differentiation, Peg-3, PP1 REGULATORY SUBUNIT, Ppp1r15a	protein phosphatase 1, regulatory (inhibitor) subunit 15A	202014_at
RARB	A830025K23, BETA RAR, HAP, LOC51036, NR1B2, RAR BETA, RAR-EPSILON, RRB2	retinoic acid receptor, beta	208530_s_at
TEK	AA517024, CD202B, Hyk, MGC139569, TIE-2, VMCM, VMCM1	TEK tyrosine kinase, endothelial	206702_at
TTF1	AV245725, RGD1565673, Ttf-I	transcription termination factor, RNA polymerase I	204772_s_at
USF2	bHLHb12, FIP, MGC91056	upstream transcription factor 2, c-fos interacting	202152_x_at

**Table 4 pone-0008250-t004:** The 25 cancer related genes of the 100 features selected by LOOCSFS on original training group of MAQC-II breast cancer data for pCR prediction.

Symbol	Synonym(s)	Entrez Gene Name	Affymetrix
ADAM17	Alpha Secretase, CD156b, cSVP, MGC71942, TACE, TNFA CONVERTASE	ADAM metallopeptidase domain 17	213532_at
APP	A beta 25–35, A-BETA 40, A-BETA 42, AAA, ABETA, ABPP, AD1, Adap, AL024401, AMYLOID BETA, AMYLOID BETA 40, AMYLOID BETA 40 HUMAN, AMYLOID BETA 42, Amyloid beta A4, AMYLOID BETA PEPTIDE 40, Amyloid precursor, Amyloidogenic glycoprotein, App alpha, APPI, appican, BETAAPP, CTFgamma, CVAP, E030013M08RIK, Nexin II, P3, PN2, PreA4, PROTEASE NEXIN2	amyloid beta (A4) precursor protein	214953_s_at
ARAF	1200013E08Rik, ARAF1, AW495444, PKS, PKS2, RAFA1	v-raf murine sarcoma 3611 viral oncogene homolog	201895_at
CEACAM1	bb-1, BGP, BGP1, BGPA, Bgpd, BGPI, BGPR, C-CAM, C-CAM1, CCAM105, CD66, CD66A, Cea-1, Cea-7, CEACAM1-4L, ECTO ATPASE, HV2, mCEA1, Mhv-1, MHVR, MHVR1, mmCGM1, mmCGM1a, mmCGM2, Pp120	carcinoembryonic antigen-related cell adhesion molecule 1 (biliary glycoprotein)	211889_x_at
CTSL2	1190035F06Rik, Cathepsin l, CATHEPSIN V, CATHL, CATL2, Ctsl, Ctsl1, CTSU, CTSV, fs, MEP, MGC125957, nkt	cathepsin L2	210074_at
CYR61	AI325051, CCN1, Cysteine-rich protein 61, GIG1, IGFBP10, MGC93040	cysteine-rich, angiogenic inducer, 61	210764_s_at
DKK1	Dkk1 predicted, mdkk-1, SK	dickkopf homolog 1 (Xenopus laevis)	204602_at
DRD2	D2, D2 DOPAMINE RECEPTOR, D2a dopamine receptor, D2DR, D2R, D2S, DOPAMINE D2 RECEPTOR, Dr2	dopamine receptor D2	216924_s_at
ETS2	AU022856, ETS2IT1	v-ets erythroblastosis virus E26 oncogene homolog 2 (avian)	201329_s_at
GRIA2	GLUR-B, GluR-K2, GLUR2, GLUR2 IONOTROPIC, HBGR2	glutamate receptor, ionotropic, AMPA 2	205358_at
ITGB4	AA407042, C230078O20, CD104, INTEGRIN-BETA 4	integrin, beta 4	204990_s_at
KIF3A	111-11-71, 111-11-86, AF180004, AF180009, AW124694, KIF3, Kifl, Kns3	kinesin family member 3A	213623_at
KLF6	Aa1017, AI448727, BCD1, C86813, COPEB, CPBP, DKFZp686N0199, Erythropoietin 1, FM2, FM6, GBF, Ierepo1, IEREPO3, KRUPPEL LIKE ZINC FINGER PROTEIN ZF9, PAC1, PROTO-ONCOGENE BCD, Proto-oncogene BCD1, R75280, ST12, ZF9	Kruppel-like factor 6	211610_at
LTBP1	9430031G15Rik, 9830146M04, MGC163161, TGFB	latent transforming growth factor beta binding protein 1	202729_s_at
MCF2	B230117G22Rik, DBL, MGC159138, RGD1566098	MCF.2 cell line derived transforming sequence	208017_s_at
REST	2610008J04RIK, AA407358, D14MGI11, MGC150099, NRSF, XBR	RE1-silencing transcription factor	204535_s_at
SDC1	AA408134, AA409076, BB4, CD138, HSPG, SDC, SYN1, Synd, SYND1, SYNDECA, Syndecan, SYNDECAN-1	syndecan 1	201287_s_at
SEMA3B	AW208495, FLJ34863, LUCA-1, SEMA, SEMA5, SEMAA, semaV	sema domain, immunoglobulin domain (Ig), short basic domain, secreted, (semaphorin) 3B	203071_at
SLPI	ALK1, ALP, ANTILEUKOPROTEASE, antileukoproteinase, BLPI, HUSI, HUSI-I, MPI, Secretory Leukoprotease Inhibitor, SLP1, WAP4, WFDC4	secretory leukocyte peptidase inhibitor	203021_at
SP1	1110003E12RIK, AA450830, AI845540, Sp1 (trans spliced isoform), SP1-1, Trans-acting transcription factor 1	Sp1 transcription factor	214732_at
TCF7L2 (includes EG:6934)	LOC679869, mTcf-4B, mTcf-4E, TCF-4, TCF4B, TCF4E, Tcf7l2	transcription factor 7-like 2 (T-cell specific, HMG-box)	216511_s_at
TNFAIP3	A20, MAD6, MGC104522, MGC138687, MGC138688, OTUD7C, TNF ALPHA-INDUCED PROTEIN 3, TNF-inducible early response, TNFA1P2, Tnfip3	tumor necrosis factor, alpha-induced protein 3	202644_s_at
TTF1	AV245725, RGD1565673, Ttf-I	transcription termination factor, RNA polymerase I	204772_s_at
TXN	ADF, AW550880, DKFZp686B1993, EOSINOPHIL CYTOTOXICITY FACTOR, MGC151960, MGC61975, THIOREDOXIN, TRX, TRX1, Txn1	thioredoxin	208864_s_at

**Table 5 pone-0008250-t005:** The 20 cancer related genes of the 100 features selected by SVMRFE on original training group of MAQC-II breast cancer data for pCR prediction.

Symbol	Synonym(s)	Entrez Gene Name	Affymetrix
APP	A beta 25–35, A-BETA 40, A-BETA 42, AAA, ABETA, ABPP, AD1, Adap, AL024401, AMYLOID BETA, AMYLOID BETA 40, AMYLOID BETA 40 HUMAN, AMYLOID BETA 42, Amyloid beta A4, AMYLOID BETA PEPTIDE 40, Amyloid precursor, Amyloidogenic glycoprotein, App alpha, APPI, appican, BETAAPP, CTFgamma, CVAP, E030013M08RIK, Nexin II, P3, PN2, PreA4, PROTEASE NEXIN2	amyloid beta (A4) precursor protein	214953_s_at
CLEC3B	DKFZp686H17246, TETRANECTIN, TN, TNA, TTN	C-type lectin domain family 3, member B	205200_at
CXCL9	BB139920, CMK, crg-10, Humig, MGC105312, MIG, SCYB9	chemokine (C-X-C motif) ligand 9	203915_at
DKK1	Dkk1 predicted, mdkk-1, SK	dickkopf homolog 1 (Xenopus laevis)	204602_at
DRD2	D2, D2 DOPAMINE RECEPTOR, D2a dopamine receptor, D2DR, D2R, D2S, DOPAMINE D2 RECEPTOR, Dr2	dopamine receptor D2	216924_s_at
EPOR	EP-R, ERYTHROPOIETIN RECEPTOR, MGC108723, MGC138358	erythropoietin receptor	215054_at
ESR1	AA420328, Alpha estrogen receptor, AU041214, DKFZp686N23123, ER, ER ALPHA, Er alpha (46 kDa isoform), ER66, ERA, ER[a], ESR, ESRA, ESTR, ESTRA, ESTROGEN RECEPTOR ALPHA, ESTROGEN RECEPTOR1, NR3A1, RNESTROR, TERP-1	estrogen receptor 1	215552_s_at
FAM153A	KIAA0752, NY-REN-7	family with sequence similarity 153, member A	211166_at
GSN	DKFZp313L0718, GELSOLIN, MGC28083, MGC95032	gelsolin (amyloidosis, Finnish type)	214040_s_at
IFNAR1	ALPHA CHAIN OF TYPE I IFNR, AVP, BETA R1, CD118, Ifar, IFN RECEPTOR TYPE 1, IFN TYPE 1 RECEPTOR, IFN-alpha-beta-R, IFN-ALPHA-REC, IFNalpha/betaR, IFNAR, IFNBR, IFRC, Infar, INFAR1, Interferon Receptor, LOC284829, Type I infr	interferon (alpha, beta and omega) receptor 1	204191_at
KL	ALPHA KLOTHO, alpha-kl, KLOTHO	klotho	205978_at
NAIP	AV364616, BIRC1, BIRC1A, Birc1b, Birc1e, BIRC1F, D13Lsd1, FLJ18088, FLJ42520, FLJ58811, LGN1, LOC652755, Naip-rs1, Naip-rs3, Naip-rs4, Naip-rs4A, Naip1, Naip2, Naip5, Naip6, NLRB1, psiNAIP, RGD1559914	NLR family, apoptosis inhibitory protein	204861_s_at
NDST1	1200015G06RIK, HSNST, HSST, HSST1, NST1	N-deacetylase/N-sulfotransferase (heparan glucosaminyl) 1	202608_s_at
PPP1R15A (includes EG:23645)	9630030H21, GADD34, MYD116, Myeloid Differentiation, Peg-3, PP1 REGULATORY SUBUNIT, Ppp1r15a	protein phosphatase 1, regulatory (inhibitor) subunit 15A	202014_at
PTGIS	CYP8, CYP8A1, MGC126858, MGC126860, Pcs, Pgi2, PGIS, PROSTACYCLIN, Prostacyclin Synthase, PTGI	prostaglandin I2 (prostacyclin) synthase	211892_s_at
RAD23B	0610007D13Rik, AV001138, HHR23B, HR23B, MGC112630, mHR23B, P58	RAD23 homolog B (S. cerevisiae)	201223_s_at
SP1	1110003E12RIK, AA450830, AI845540, Sp1 (trans spliced isoform), SP1-1, Trans-acting transcription factor 1	Sp1 transcription factor	214732_at
TACSTD2	C80403, EGP-1, GA733, GA733-1, Ly97, M1S1, MGC141612, MGC141613, MGC72570, Prp1, TROP2	tumor-associated calcium signal transducer 2	202286_s_at
TUBB3	3200002H15Rik, beta-4, M(beta)3, M(beta)6, MC1R, Nst, Tub beta3, TUBB4, Tubulin beta-3, Tubulin beta-III, Tuj1	tubulin, beta 3	202154_x_at
ZNF10	KOX1	zinc finger protein 10	216350_s_at

**Table 6 pone-0008250-t006:** The 34 cancer related genes of the 100 features selected by NBC-MSC on original training group of MAQC-II breast cancer data for erpos prediction.

Symbol	Synonym(s)	Entrez Gene Name	Affymetrix
APP	A beta 25–35,A-BETA 40,A-BETA 42,AAA,ABETA,ABPP,AD1,Adap,AL024401,AMYLOID BETA,AMYLOID BETA 40,AMYLOID BETA 40 HUMAN,AMYLOID BETA 42,Amyloid beta A4,AMYLOID BETA PEPTIDE 40,Amyloid precursor,Amyloidogenic glycoprotein,App alpha,APPI,appican,BETAAPP,CTFgamma,CVAP,E030013M08RIK,Nexin II,P3,PN2,PreA4,PROTEASE NEXIN2	amyloid beta (A4) precursor protein	214953_s_at
CCDC28A	1700009P13Rik,AI480677,C6ORF80,CCRL1AP,DKFZP586D0623,MGC116395,MGC131913,MGC19351,RGD1310326	coiled-coil domain containing 28A	209479_at
CEACAM1	bb-1,BGP,BGP1,BGPA,Bgpd,BGPI,BGPR,C-CAM,C-CAM1,CCAM105,CD66,CD66A,Cea-1,Cea-7,CEACAM1-4L,ECTO ATPASE,HV2,mCEA1,Mhv-1,MHVR,MHVR1,mmCGM1,mmCGM1a,mmCGM2,Pp120	carcinoembryonic antigen-related cell adhesion molecule 1 (biliary glycoprotein)	211883_x_at
CHRNB4	Acrb-4,NACHR BETA4	cholinergic receptor, nicotinic, beta 4	207516_at
CXCL9	BB139920,CMK,crg-10,Humig,MGC105312,MIG,SCYB9	chemokine (C-X-C motif) ligand 9	203915_at
EIF1	A121,EIF1A,ISO1,MGC101938,MGC6503,SUI1,SUI1-RS1	eukaryotic translation initiation factor 1	212130_x_at
EMP1	CL-20,ENP1MR,EPITHELIAL MEMBRANE PROTEIN 1,MGC93627,TMP	epithelial membrane protein 1	201325_s_at
EPOR	EP-R,ERYTHROPOIETIN RECEPTOR,MGC108723,MGC138358	erythropoietin receptor	215054_at
ETV5	1110005E01Rik,8430401F14Rik,ERM	ets variant 5	203349_s_at
FBLN1	FBLN,FIBULIN 1	fibulin 1	207834_at
FBXL7	AL023057,D230018M15Rik,FBL6,FBL7,FBP7,MGC102204	F-box and leucine-rich repeat protein 7	213249_at
GHR	AA986417,GHBP,GHR/BP,GROWTH HORMONE RECEPTOR,MGC124963,MGC156665	growth hormone receptor	205498_at
GPC3	DGSX,Glypican 3,GTR2-2,MGC93606,OCI-5,SDYS,SGB,SGBS,SGBS1	glypican 3	209220_at
GPS2	AI505953,AMF-1,MGC104294,MGC119287,MGC119288,MGC119289	G protein pathway suppressor 2	209350_s_at
IGF2BP3	2610101N11Rik,AA522010,Ab2-255,AL022933,AU045931,DKFZp686F1078,IMP-3,KOC1,Koc13,mimp3,Neilsen,RGD1306512,VICKZ3	insulin-like growth factor 2 mRNA binding protein 3	203820_s_at
ING4	D6Wsu147e,D6Xrf92,MGC12557,MGC156688,my036,p29ING4,p33ING1 ISOLOG	inhibitor of growth family, member 4	218234_at
ITGB4	AA407042,C230078O20,CD104,INTEGRIN-BETA 4	integrin, beta 4	204989_s_at
KLF5	4930520J07Rik,BTEB2,CKLF,IKLF,Kruppel-like factor 5,mBTEB2	Kruppel-like factor 5 (intestinal)	209211_at
MFAP3L	4933428A15RIK,5430405D20Rik,AI461995,AW125052,KIAA0626,mKIAA0626,NYD-sp9	microfibrillar-associated protein 3-like	210493_s_at
NCOR1	5730405M06RIK,A230020K14RIK,hCIT529I10,hN-CoR,KIAA1047,MGC104216,MGC116328,mKIAA1047,N-COR,RIP13,Rxrip13,TRAC1	nuclear receptor co-repressor 1	200854_at
PA2G4	38kDa,AA672939,EBP1,HG4-1,Itaf45,MGC94070,p38-2G4,Plfap,PROLIFERATION ASSOCIATED 2G4,Proliveration-associated protein 1	proliferation-associated 2G4, 38kDa	214794_at
PCNA	MGC8367,Pcna/cyclin,PCNAR	proliferating cell nuclear antigen	217400_at
PLD1	AA536939,C85393,Pc-Plc,Phospholipase D1,PLD1A,PLD1B,Plda,Pldb	phospholipase D1, phosphatidylcholine-specific	215723_s_at
RPL13A	1810026N22Rik,23-KD HIGHLY BASIC,MGC107571,Ribol13a,Ribosomal protein l13a,Tstap198-7,tum-antigen	ribosomal protein L13a	211942_x_at
SCGB1D2	BU101,LIPB,LIPOPHILIN B,LPHB	secretoglobin, family 1D, member 2	206799_at
SEL1L	AW493766,IBD2,KIAA4137,mKIAA4137,PRO1063,SEL1,SEL1-LIKE,SEL1H	sel-1 suppressor of lin-12-like (C. elegans)	202063_s_at
SERPINA6	AI265318,AV104445,CBG,MGC112780	serpin peptidase inhibitor, clade A (alpha-1 antiproteinase, antitrypsin), member 6	206325_at
SMA4	b55C20.2,FLJ36702,MGC22265,MGC60382,SMA3	glucuronidase, beta pseudogene	214850_at
SMC4	2500002A22Rik,C79747,CAP-C,DKFZP434F205,HCAP-C,MGC125078,SMC4L1	structural maintenance of chromosomes 4	215623_x_at
SOS1	9630010N06,AI449023,GF1,GGF1,GINGF,HGF,MSOS1,NS4,Sos	son of sevenless homolog 1 (Drosophila)	212780_at
TNFRSF11A	CD265,FEO,LOH18CR1,Ly109,MGC112793,mRANK,ODAR,ODFR,OFE,OPGL receptor,OPTB7,OSTS,PDB2,RANK,RGD1563614,TRANCE-R	tumor necrosis factor receptor superfamily, member 11a, NFKB activator	207037_at
TNFSF12	APO3L,DR3L,DR3LG,MGC129581,MGC20669,TWEAK	tumor necrosis factor (ligand) superfamily, member 12	205611_at
TRPV6	ABP/ZF,Cac,CAT,CAT-L,CAT1,Crac,ECAC2,HSA277909,LP6728,Otrpc3,ZFAB	transient receptor potential cation channel, subfamily V, member 6	206827_s_at
USP7	2210010O09Rik,AA409944,AA617399,AU019296,AW548146,C80752,HAUSP,TEF1	ubiquitin specific peptidase 7 (herpes virus-associated)	222032_s_at

**Table 7 pone-0008250-t007:** The 33 cancer related genes of the 100 features selected by NMSC-MSC on original training group of MAQC-II breast cancer data for erpos prediction.

Symbol	Synonym(s)	Entrez Gene Name	Affymetrix
BAG1	BAG1L, Bag1s, RAP46	BCL2-associated athanogene	202387_at
CCDC28A	1700009P13Rik, AI480677, C6ORF80, CCRL1AP, DKFZP586D0623, MGC116395, MGC131913, MGC19351, RGD1310326	coiled-coil domain containing 28A	209479_at
CEACAM1	bb-1, BGP, BGP1, BGPA, Bgpd, BGPI, BGPR, C-CAM, C-CAM1, CCAM105, CD66, CD66A, Cea-1, Cea-7, CEACAM1-4L, ECTO ATPASE, HV2, mCEA1, Mhv-1, MHVR, MHVR1, mmCGM1, mmCGM1a, mmCGM2, Pp120	carcinoembryonic antigen-related cell adhesion molecule 1 (biliary glycoprotein)	211883_x_at
CHRNB4	Acrb-4, NACHR BETA4	cholinergic receptor, nicotinic, beta 4	207516_at
EIF1	A121, EIF1A, ISO1, MGC101938, MGC6503, SUI1, SUI1-RS1	eukaryotic translation initiation factor 1	212130_x_at
EPOR	EP-R, ERYTHROPOIETIN RECEPTOR, MGC108723, MGC138358	erythropoietin receptor	215054_at
FBLN1	FBLN, FIBULIN 1	fibulin 1	207834_at
GBP1 (includes EG:2633)	5830475C06, GBP1, GBPI, IFI67-K, MAG-1, MGC124334, Mpa-1	guanylate binding protein 1, interferon-inducible, 67kDa	202270_at
GHR	AA986417, GHBP, GHR/BP, GROWTH HORMONE RECEPTOR, MGC124963, MGC156665	growth hormone receptor	205498_at
GPC3	DGSX, Glypican 3, GTR2-2, MGC93606, OCI-5, SDYS, SGB, SGBS, SGBS1	glypican 3	209220_at
GPS2	AI505953, AMF-1, MGC104294, MGC119287, MGC119288, MGC119289	G protein pathway suppressor 2	209350_s_at
IMPDH2	ENSMUSG00000071041, Imp dehydrogenase 2, IMPD, IMPD2, Impdh, IMPDH-II, inosine 5′-phosphate dehydrogenase 2, MGC72938, OTTMUSG00000019498	IMP (inosine monophosphate) dehydrogenase 2	201892_s_at
ING4	D6Wsu147e, D6Xrf92, MGC12557, MGC156688, my036, p29ING4, p33ING1 ISOLOG	inhibitor of growth family, member 4	218234_at
ITGB4	AA407042, C230078O20, CD104, INTEGRIN-BETA 4	integrin, beta 4	204990_s_at
KLF5	4930520J07Rik, BTEB2, CKLF, IKLF, Kruppel-like factor 5, mBTEB2	Kruppel-like factor 5 (intestinal)	209211_at
MAP3K12	DLK, MUK, PK, ZPK, ZPKP1	mitogen-activated protein kinase kinase kinase 12	205447_s_at
MFAP3L	4933428A15RIK, 5430405D20Rik, AI461995, AW125052, KIAA0626, mKIAA0626, NYD-sp9	microfibrillar-associated protein 3-like	210493_s_at
NCOR1	5730405M06RIK, A230020K14RIK, hCIT529I10, hN-CoR, KIAA1047, MGC104216, MGC116328, mKIAA1047, N-COR, RIP13, Rxrip13, TRAC1	nuclear receptor co-repressor 1	200854_at
PA2G4	38kDa, AA672939, EBP1, HG4-1, Itaf45, MGC94070, p38-2G4, Plfap, PROLIFERATION ASSOCIATED 2G4, Proliveration-associated protein 1	proliferation-associated 2G4, 38kDa	214794_at
PCNA	MGC8367, Pcna/cyclin, PCNAR	proliferating cell nuclear antigen	217400_at
PLA2G16	ADPLA, C78643, H-REV107-1, HRASLS3, HREV107, HREV107-3, MGC118754	phospholipase A2, group XVI	209581_at
PLD1	AA536939, C85393, Pc-Plc, Phospholipase D1, PLD1A, PLD1B, Plda, Pldb	phospholipase D1, phosphatidylcholine-specific	215723_s_at
PTPN14	C130080N23RIK, MGC126803, OTTMUSG00000022087, PEZ, PTP36, PTPD2	protein tyrosine phosphatase, non-receptor type 14	205503_at
SCGB1D2	BU101, LIPB, LIPOPHILIN B, LPHB	secretoglobin, family 1D, member 2	206799_at
SEL1L	AW493766, IBD2, KIAA4137, mKIAA4137, PRO1063, SEL1, SEL1-LIKE, SEL1H	sel-1 suppressor of lin-12-like (C. elegans)	202063_s_at
SERPINA6	AI265318, AV104445, CBG, MGC112780	serpin peptidase inhibitor, clade A (alpha-1 antiproteinase, antitrypsin), member 6	206325_at
SMC4	2500002A22Rik, C79747, CAP-C, DKFZP434F205, HCAP-C, MGC125078, SMC4L1	structural maintenance of chromosomes 4	215623_x_at
SPAM1 (includes EG:6677)	4933439A12Rik, HYA1, HYAL1, HYAL3, HYAL5, MGC108951, MGC26532, PH-20, SPAG15, SPAM1, TESTICULAR HYALURONIDASE	sperm adhesion molecule 1 (PH-20 hyaluronidase, zona pellucida binding)	210536_s_at
TCF3	A1, AA408400, ALF2, AW209082, bHLHb21, E12, E12/E47, E2-5, E2A, E47, ITF1, ME2, MGC129647, MGC129648, PAN1, Pan2, Tcfe2a, TRANSCRIPTION FACTOR 3, VDIR	transcription factor 3 (E2A immunoglobulin enhancer binding factors E12/E47)	213730_x_at
TGFA	RATTGFAA, TFGA, TGF ALPHA, TGFAA, wa-1	transforming growth factor, alpha	211258_s_at
TNFRSF11A	CD265, FEO, LOH18CR1, Ly109, MGC112793, mRANK, ODAR, ODFR, OFE, OPGL receptor, OPTB7, OSTS, PDB2, RANK, RGD1563614, TRANCE-R	tumor necrosis factor receptor superfamily, member 11a, NFKB activator	207037_at
TNFSF12	APO3L, DR3L, DR3LG, MGC129581, MGC20669, TWEAK	tumor necrosis factor (ligand) superfamily, member 12	205611_at
USP7	2210010O09Rik, AA409944, AA617399, AU019296, AW548146, C80752, HAUSP, TEF1	ubiquitin specific peptidase 7 (herpes virus-associated)	222032_s_at

**Table 8 pone-0008250-t008:** The 40 cancer related genes of the 100 features selected by GLGS on original training group of MAQC-II breast cancer data for erpos prediction.

Symbol	Synonym(s)	Entrez Gene Name	Affymetrix
ADRA2A	ADRA-2, ADRA2R, ADRAR, ADRENOCEPTOR ALPHA2A, alpha(2A)AR, ALPHA-2A ADRENERGIC RECEPTOR, ALPHA2-C10, alpha2A, Alpha2a Adrenoceptor, ALPHA2A-AR, AW122659, RATRG20, RG20, ZNF32	adrenergic, alpha-2A-, receptor	209869_at
ANGPTL4	ANGIOPOIETIN-LIKE 4, ANGPTL2, ARP4, Bk89, Fasting Induced Adipose Factor, FIAF, Harp, HFARP, LOC362850, Ng27, NL2, PGAR, PGARG, pp1158, PPARG	angiopoietin-like 4	221009_s_at
BAG1	BAG1L, Bag1s, RAP46	BCL2-associated athanogene	202387_at
CCNE1	AW538188, CCNE, CYCLE, CYCLIN E, Cyclin E1	cyclin E1	213523_at
CHRNA5	AChR alpha5, Acra-5, ALPHA5 NACHR, ALPHA5 NICOTINIC RECEPTOR, LNCR2, MGC124059, MGC124168, nAChR alpha5, sialoprotein	cholinergic receptor, nicotinic, alpha 5	206533_at
CHRNB4	Acrb-4, NACHR BETA4	cholinergic receptor, nicotinic, beta 4	207516_at
CTSL2	1190035F06Rik, Cathepsin l, CATHEPSIN V, CATHL, CATL2, Ctsl, Ctsl1, CTSU, CTSV, fs, MEP, MGC125957, nkt	cathepsin L2	210074_at
EGFR	9030024J15RIK, AI552599, EGF RECEPTOR, EGF-TK, EGFR1, EGFRec, ER2, ERBB, ERBB1, Errp, HER1, MENA, PIG61, wa-2, Wa5	epidermal growth factor receptor (erythroblastic leukemia viral (v-erb-b) oncogene homolog, avian)	201983_s_at
EPHA2	AW545284, ECK, ECKR, Myk2, Sek-2	EPH receptor A2	203499_at
EPOR	EP-R, ERYTHROPOIETIN RECEPTOR, MGC108723, MGC138358	erythropoietin receptor	215054_at
FBLN1	FBLN, FIBULIN 1	fibulin 1	207834_at
GPC3	DGSX, Glypican 3, GTR2-2, MGC93606, OCI-5, SDYS, SGB, SGBS, SGBS1	glypican 3	209220_at
GPS2	AI505953, AMF-1, MGC104294, MGC119287, MGC119288, MGC119289	G protein pathway suppressor 2	209350_s_at
HMGA1	AL023995, HMG-I(Y), HMG-R, Hmg-y/i, HMGA1A, Hmgi, Hmgi/y, HMGIY, HMGY, MGC102580, MGC12816, MGC4242, MGC4854	high mobility group AT-hook 1	206074_s_at
HNRPDL	AA407431, AA959857, D5Ertd650e, D5Wsu145e, HNRNP, HNRNP-D LIKE, hnRNP-DL, JKTBP, JKTBP1, JKTBP2, laAUF1, MGC125262	heterogeneous nuclear ribonucleoprotein D-like	209068_at
HSD17B2	17Hsd, AI194836, AI194967, AI255511, EDH17B2, HSD17, SDR9C2	hydroxysteroid (17-beta) dehydrogenase 2	204818_at
ICAM1	BB2, CD54, ICAM, INTERCELLULAR ADHESION MOLECULE 1, Ly-47, M90551, MALA-2, Melanoma Progression Associated Antigen, MGC6195, P3.58	intercellular adhesion molecule 1	202637_s_at
IGF2BP3	2610101N11Rik, AA522010, Ab2-255, AL022933, AU045931, DKFZp686F1078, IMP-3, KOC1, Koc13, mimp3, Neilsen, RGD1306512, VICKZ3	insulin-like growth factor 2 mRNA binding protein 3	203820_s_at
ITGB4	AA407042, C230078O20, CD104, INTEGRIN-BETA 4	integrin, beta 4	204989_s_at
MAP3K12	DLK, MUK, PK, ZPK, ZPKP1	mitogen-activated protein kinase kinase kinase 12	205447_s_at
MFAP3L	4933428A15RIK, 5430405D20Rik, AI461995, AW125052, KIAA0626, mKIAA0626, NYD-sp9	microfibrillar-associated protein 3-like	210493_s_at
PCM1	2600002H09Rik, 9430077F19Rik, C030044G17Rik, LOC100044052, MGC170660, PTC4	pericentriolar material 1	214118_x_at
PCNA	MGC8367, Pcna/cyclin, PCNAR	proliferating cell nuclear antigen	217400_at
PLD1	AA536939, C85393, Pc-Plc, Phospholipase D1, PLD1A, PLD1B, Plda, Pldb	phospholipase D1, phosphatidylcholine-specific	215723_s_at
PML	1200009E24Rik, AI661194, MYL, PP8675, Retinoic acid receptor, RGD1562602, RNF71, TRIM19	promyelocytic leukemia	211013_x_at
PPP3CB	1110063J16Rik, Calcineurin A Beta, CALCINEURIN A1, CALNA2, CALNB, CnA-gamma, CnAbeta, Protein phosphatase 3 catalytic subunit beta isoform	protein phosphatase 3 (formerly 2B), catalytic subunit, beta isoform	202432_at
PURA	CAGER-1, PUR-ALPHA, PUR1, ssCRE-BP, VACSSBF1	purine-rich element binding protein A	204020_at
PVR	3830421F03Rik, CD155, D7ERTD458E, FLJ25946, HVED, mE4, NECL5, NECTIN 2 ALPHA, NECTIN-2, Pe4, PVS, TAA1, TAGE4	poliovirus receptor	212662_at
RBM5	D030069N10RIK, FLJ39876, G15, H37, LUCA15, RMB5	RNA binding motif protein 5	201395_at
SEL1L	AW493766, IBD2, KIAA4137, mKIAA4137, PRO1063, SEL1, SEL1-LIKE, SEL1H	sel-1 suppressor of lin-12-like (C. elegans)	202063_s_at
SERPINA6	AI265318, AV104445, CBG, MGC112780	serpin peptidase inhibitor, clade A (alpha-1 antiproteinase, antitrypsin), member 6	206325_at
SLC5A3	AA623876, BF642829, SMIT, Smit1, SMIT2	solute carrier family 5 (sodium/myo-inositol cotransporter), member 3	213164_at
SLC7A5	4f2 light chain, 4F2LC, CD98, CD98 LIGHT CHAIN, CD98LC, D0H16S474E, D16S469E, E16, E16/TA1, hLAT1, LAT1, MPE16, TA1	solute carrier family 7 (cationic amino acid transporter, y+ system), member 5	201195_s_at
SMC4	2500002A22Rik, C79747, CAP-C, DKFZP434F205, HCAP-C, MGC125078, SMC4L1	structural maintenance of chromosomes 4	215623_x_at
SOS1	9630010N06, AI449023, GF1, GGF1, GINGF, HGF, MSOS1, NS4, Sos	son of sevenless homolog 1 (Drosophila)	212780_at
TGFA	RATTGFAA, TFGA, TGF ALPHA, TGFAA, wa-1	transforming growth factor, alpha	205016_at
TNFRSF11A	CD265, FEO, LOH18CR1, Ly109, MGC112793, mRANK, ODAR, ODFR, OFE, OPGL receptor, OPTB7, OSTS, PDB2, RANK, RGD1563614, TRANCE-R	tumor necrosis factor receptor superfamily, member 11a, NFKB activator	207037_at
UBE2B	17-kDa Ubiquitin-Conjugating Enzyme E2, 2610301N02RIK, E2-14K, E2-17kDa, E2b, HHR6B, HR6B, LOC81816, RAD6B, UBC2	ubiquitin-conjugating enzyme E2B (RAD6 homolog)	211763_s_at
USP7	2210010O09Rik, AA409944, AA617399, AU019296, AW548146, C80752, HAUSP, TEF1	ubiquitin specific peptidase 7 (herpes virus-associated)	222032_s_at
VAMP2	FLJ11460, mVam2, RATVAMPB, RATVAMPIR, SYB, SYB2, SYNAPTOBREVIN 2, Vamp ii	vesicle-associated membrane protein 2 (synaptobrevin 2)	201557_at

**Table 9 pone-0008250-t009:** The 44 cancer related genes of the 100 features selected by LOOCSFS on original training group of MAQC-II breast cancer data for erpos prediction.

Symbol	Synonym(s)	Entrez Gene Name	Affymetrix
ACP1	4632432E04Rik, AI427468, HAAP, LMPTP, LMW-PTP, MGC103115, MGC111030, MGC132904, MGC3499	acid phosphatase 1, soluble	215227_x_at
BAG1	BAG1L, Bag1s, RAP46	BCL2-associated athanogene	202387_at
BCL2	AW986256, Bcl2 alpha, C430015F12Rik, CED9, D630044D05RIK, D830018M01RIK, LOC100046608, ORF16	B-cell CLL/lymphoma 2	203685_at
BTG2	AA959598, Agl, An, an-1, APRO1, B-cell translocation gene 2, antiproliferative, MGC126063, MGC126064, PC3, TIS21	BTG family, member 2	201236_s_at
CCDC28A	1700009P13Rik, AI480677, C6ORF80, CCRL1AP, DKFZP586D0623, MGC116395, MGC131913, MGC19351, RGD1310326	coiled-coil domain containing 28A	209479_at
CCNA2	AA408589, CCN1, CCNA, CYCA, CYCLIN A2, CYCLIN-A, MGC156527, p60	cyclin A2	213226_at
CD47	9130415E20RIK, AA407862, AI848868, AW108519, B430305P08RIK, CD47 ANTIGEN, CDw149, IAP, Itgp, Locuslink 71587, MER6, MGC93490, OA3	CD47 molecule	211075_s_at
CFD	ADIPSIN, ADN, Complement Factor D, DF, EVE, FACTOR D, PFD	complement factor D (adipsin)	205382_s_at
CHRNA5	AChR alpha5, Acra-5, ALPHA5 NACHR, ALPHA5 NICOTINIC RECEPTOR, LNCR2, MGC124059, MGC124168, nAChR alpha5, sialoprotein	cholinergic receptor, nicotinic, alpha 5	206533_at
CTSL2	1190035F06Rik, Cathepsin l, CATHEPSIN V, CATHL, CATL2, Ctsl, Ctsl1, CTSU, CTSV, fs, MEP, MGC125957, nkt	cathepsin L2	210074_at
CXCL10	C7, CRG-2, GAMMA-IFN INDUCIBLE EARLY RESPONSE, gIP-10, IFI10, IFNG INDUCIBLE PROTEIN-10, INP10, Interferon-inducible protein-10, IP-10, mob-1, SCYB10, SMALL INDUCIBLE CYTOKINE SUBFAMILY B (Cys-X-Cys), MEMBER 10	chemokine (C-X-C motif) ligand 10	204533_at
CYB5A	0610009N12Rik, CB5, CYB5, Cytb5, CYTOCHROME B5, MCB5, MGC108694, MGC128769	cytochrome b5 type A (microsomal)	209366_x_at
DDX17	2610007K22RIK, A430025E01Rik, AI047725, C80929, DEAD-box protein p72, DEAD/H box RNA helicase p72, DKFZp761H2016, Gm926, MGC109323, MGC79147, P72, RH70	DEAD (Asp-Glu-Ala-Asp) box polypeptide 17	208718_at
DNMT3B	DNA MTase HsaIIIB, ICF, M.HsaIIIB, MGC124407	DNA (cytosine-5-)-methyltransferase 3 beta	220668_s_at
EPHA2	AW545284, ECK, ECKR, Myk2, Sek-2	EPH receptor A2	203499_at
ERBB4	C-erb-b4, Erbb4 Cyt2, ERBB4 JM-A, HER4, MGC138404, p180erbB4	v-erb-a erythroblastic leukemia viral oncogene homolog 4 (avian)	214053_at
ESR1	AA420328, Alpha estrogen receptor, AU041214, DKFZp686N23123, ER, ER ALPHA, Er alpha (46 kDa isoform), ER66, ERA, ER[a], ESR, ESRA, ESTR, ESTRA, ESTROGEN RECEPTOR ALPHA, ESTROGEN RECEPTOR1, NR3A1, RNESTROR, TERP-1	estrogen receptor 1	205225_at
ETV5	1110005E01Rik, 8430401F14Rik, ERM	ets variant 5	203349_s_at
FAM134B	1810015C04RIK, AU015349, FLJ20152, FLJ22155, FLJ22179	family with sequence similarity 134, member B	218510_x_at
FBLN1	FBLN, FIBULIN 1	fibulin 1	207834_at
GREB1	5730583K22Rik, 9130004E13, AF180470, AU023194, KIAA0575, mKIAA0575	GREB1 protein	205862_at
IL6ST	5133400A03Rik, AA389424, Ac1055, BB405851, CD130, CDw130, D13Ertd699e, GP130, GP130-RAPS, Il6 transd, IL6R-beta	interleukin 6 signal transducer (gp130, oncostatin M receptor)	211000_s_at
IRS1	ENSMUSG00000022591, G972R, HIRS-1, IRS1IRM	insulin receptor substrate 1	204686_at
ITPR1	D6Pas2, I145TR, InsP3R, INSP3R1, IP3 RECEPTOR, Ip3 Receptor Type 1, IP3R, IP3R1, opt, P400, Pcd6, Pcp-1, SCA15, SCA16	inositol 1,4,5-triphosphate receptor, type 1	203710_at
LRP8	4932703M08Rik, AA921429, AI848122, APOER2, HSZ75190, LR8B, MCI1	low density lipoprotein receptor-related protein 8, apolipoprotein e receptor	208433_s_at
MAP3K12	DLK, MUK, PK, ZPK, ZPKP1	mitogen-activated protein kinase kinase kinase 12	205447_s_at
MAPT	AI413597, ALZ50, AW045860, DDPAC, FLJ31424, FTDP-17, MAPTL, MGC134287, MGC138549, MGC156663, MSTD, Mtapt, MTBT1, MTBT2, PHF TAU, PPND, pTau, RNPTAU, TAU, Tau 3r, Tau-1, TAU-FACTOR, Tau40, TAU4R	microtubule-associated protein tau	203929_s_at
MCM7	AI747533, CDABP0042, CDC47, D16Mgi24, mCDC47, MCM2, Mcmd7, MGC93853, P1.1-MCM3, P1CDC47, P85MCM, PNAS-146	minichromosome maintenance complex component 7	208795_s_at
MYO10	AW048724, D15Ertd600e, FLJ10639, FLJ21066, FLJ22268, FLJ43256, KIAA0799, MGC131988, mKIAA0799, Myo10 (predicted), myosin-X	myosin X	201976_s_at
NCAPG (includes EG:64151)	CAP-G, CHCG, FLJ12450, HCAP-G, MGC126525, NCAPG, NY-MEL-3, RGD1562646	non-SMC condensin I complex, subunit G	218663_at
NDRG1	CAP43, CMT4D, DRG1, GC4, HMSNL, N-myc Downstream Regulated 1, N-myc downstream regulatory protein 1, NDR1, NDRL, NMSL, PROXY1, RIT42, RTP, TARG1, TDD5	N-myc downstream regulated 1	200632_s_at
NOTCH3	AW229011, CADASIL, CASIL	Notch homolog 3 (Drosophila)	203238_s_at
NPY1R	MGC109393, NPY receptor Y1, NPY Y1 receptor, NPY-1, NPYIR, NPYR, Y1, Y1 RECEPTOR, Y1-R	neuropeptide Y receptor Y1	205440_s_at
PCM1	2600002H09Rik, 9430077F19Rik, C030044G17Rik, LOC100044052, MGC170660, PTC4	pericentriolar material 1	214118_x_at
PDZK1	1700023D20Rik, 2610507N21Rik, 4921513F16Rik, AI267131, AI314638, AL022680, CAP70, CLAMP, D3Ertd537e, mPDZK1, NHERF3, PDZD1, Sodium sulfate cotransporter	PDZ domain containing 1	205380_at
PTPN14	C130080N23RIK, MGC126803, OTTMUSG00000022087, PEZ, PTP36, PTPD2	protein tyrosine phosphatase, non-receptor type 14	205503_at
PVR	3830421F03Rik, CD155, D7ERTD458E, FLJ25946, HVED, mE4, NECL5, NECTIN 2 ALPHA, NECTIN-2, Pe4, PVS, TAA1, TAGE4	poliovirus receptor	212662_at
RPS15	EG633683, MGC111130, RIG	ribosomal protein S15	200819_s_at
RRM2	AA407299, MGC113712, MGC116120, R2, Ribonucleoside-diphosphate reductase M2 subunit, Ribonucleotide reductase non-heme subunit, RIBONUCLEOTIDE REDUCTASE SMALL SUBUNIT, Rnr-r2, RNRII, RR2, RR2M	ribonucleotide reductase M2 polypeptide	209773_s_at
SEL1L	AW493766, IBD2, KIAA4137, mKIAA4137, PRO1063, SEL1, SEL1-LIKE, SEL1H	sel-1 suppressor of lin-12-like (C. elegans)	202063_s_at
SLC39A6	Ermelin, LIV-1	solute carrier family 39 (zinc transporter), member 6	202088_at
TMBIM4	0610007H07Rik, AU022431, CGI-119, GAAP, MGC73002, S1R, ZPRO	transmembrane BAX inhibitor motif containing 4	219206_x_at
UBE2B	17-kDa Ubiquitin-Conjugating Enzyme E2, 2610301N02RIK, E2-14K, E2-17kDa, E2b, HHR6B, HR6B, LOC81816, RAD6B, UBC2	ubiquitin-conjugating enzyme E2B (RAD6 homolog)	211763_s_at
VEGFA	Gd-vegf, MGC70609, VEGF, Vegf-3, VEGF1, VEGF120, VEGF164, Vegf165, Vegf188, Vegfa 188, VPF, VPF/VEGF	vascular endothelial growth factor A	210513_s_at

**Table 10:** The 40 cancer related genes of the 100 features selected by SVMRFE on original training group of MAQC-II breast cancer data for erpos prediction.

Symbol	Synonym(s)	Entrez Gene Name	Affymetrix
ANGPTL4	ANGIOPOIETIN-LIKE 4, ANGPTL2, ARP4, Bk89, Fasting Induced Adipose Factor, FIAF, Harp, HFARP, LOC362850, Ng27, NL2, PGAR, PGARG, pp1158, PPARG	angiopoietin-like 4	221009_s_at
APP	A beta 25–35, A-BETA 40, A-BETA 42, AAA, ABETA, ABPP, AD1, Adap, AL024401, AMYLOID BETA, AMYLOID BETA 40, AMYLOID BETA 40 HUMAN, AMYLOID BETA 42, Amyloid beta A4, AMYLOID BETA PEPTIDE 40, Amyloid precursor, Amyloidogenic glycoprotein, App alpha, APPI, appican, BETAAPP, CTFgamma, CVAP, E030013M08RIK, Nexin II, P3, PN2, PreA4, PROTEASE NEXIN2	amyloid beta (A4) precursor protein	214953_s_at
CHRNB4	Acrb-4, NACHR BETA4	cholinergic receptor, nicotinic, beta 4	207516_at
CREBL2	AI046348, B230205M03, MGC109304, MGC117311, MGC130380, MGC130381, MGC138362	cAMP responsive element binding protein-like 2	201990_s_at
CTSL2	1190035F06Rik, Cathepsin l, CATHEPSIN V, CATHL, CATL2, Ctsl, Ctsl1, CTSU, CTSV, fs, MEP, MGC125957, nkt	cathepsin L2	210074_at
CX3CL1	AB030188, ABCD-3, AI848747, C3Xkine, CX3C, CX3CL, CXC3, CXC3 CHEMOKINE PRECURSOR, CXC3C, D8Bwg0439e, FK, FKN, FRACTALKINE, NEUROTACTIN, NTN, NTT, SCYD1	chemokine (C-X3-C motif) ligand 1	823_at
DACH1	AI182278, Dac, DACH, E130112M23Rik, FLJ10138	dachshund homolog 1 (Drosophila)	205471_s_at
E2F1	E2f, E2f1 predicted, KIAA4009, mKIAA4009, RBAP1, RBBP3, RBP3	E2F transcription factor 1	204947_at
EPOR	EP-R, ERYTHROPOIETIN RECEPTOR, MGC108723, MGC138358	erythropoietin receptor	215054_at
ERBB4	C-erb-b4, Erbb4 Cyt2, ERBB4 JM-A, HER4, MGC138404, p180erbB4	v-erb-a erythroblastic leukemia viral oncogene homolog 4 (avian)	214053_at
ESR1	AA420328, Alpha estrogen receptor, AU041214, DKFZp686N23123, ER, ER ALPHA, Er alpha (46 kDa isoform), ER66, ERA, ER[a], ESR, ESRA, ESTR, ESTRA, ESTROGEN RECEPTOR ALPHA, ESTROGEN RECEPTOR1, NR3A1, RNESTROR, TERP-1	estrogen receptor 1	217190_x_at
FAM152A	5830417C01Rik, C1orf121, CGI-146, DKFZp586C1019, FLJ21998, PNAS-4	family with sequence similarity 152, member A	212371_at
FBLN1	FBLN, FIBULIN 1	fibulin 1	207834_at
GATA3	HDR, MGC2346, MGC5199, MGC5445	GATA binding protein 3	209602_s_at
GHR	AA986417, GHBP, GHR/BP, GROWTH HORMONE RECEPTOR, MGC124963, MGC156665	growth hormone receptor	205498_at
GPC3	DGSX, Glypican 3, GTR2-2, MGC93606, OCI-5, SDYS, SGB, SGBS, SGBS1	glypican 3	209220_at
GREB1	5730583K22Rik, 9130004E13, AF180470, AU023194, KIAA0575, mKIAA0575	GREB1 protein	205862_at
IGF2BP3	2610101N11Rik, AA522010, Ab2-255, AL022933, AU045931, DKFZp686F1078, IMP-3, KOC1, Koc13, mimp3, Neilsen, RGD1306512, VICKZ3	insulin-like growth factor 2 mRNA binding protein 3	203820_s_at
IL6ST	5133400A03Rik, AA389424, Ac1055, BB405851, CD130, CDw130, D13Ertd699e, GP130, GP130-RAPS, Il6 transd, IL6R-beta	interleukin 6 signal transducer (gp130, oncostatin M receptor)	212195_at
IRS1	ENSMUSG00000022591, G972R, HIRS-1, IRS1IRM	insulin receptor substrate 1	204686_at
ITGB4	AA407042, C230078O20, CD104, INTEGRIN-BETA 4	integrin, beta 4	204990_s_at
ITPR1	D6Pas2, I145TR, InsP3R, INSP3R1, IP3 RECEPTOR, Ip3 Receptor Type 1, IP3R, IP3R1, opt, P400, Pcd6, Pcp-1, SCA15, SCA16	inositol 1,4,5-triphosphate receptor, type 1	211323_s_at
LMO4	A730077C12Rik, Crp3, Etohi4, MGC105593	LIM domain only 4	209205_s_at
MFAP3L	4933428A15RIK, 5430405D20Rik, AI461995, AW125052, KIAA0626, mKIAA0626, NYD-sp9	microfibrillar-associated protein 3-like	210493_s_at
MGMT	AGAT, AGT, AI267024, ATase, MGC107020, O6-ALKYLGUANINE DNA ALKYLTRANSFERASE	O-6-methylguanine-DNA methyltransferase	204880_at
PCNA	MGC8367, Pcna/cyclin, PCNAR	proliferating cell nuclear antigen	217400_at
PGR	9930019P03Rik, BB114106, ENSMUSG00000074510, LOC360433, NR3C3, Pgrb, PR, PR BETA, PR-A, PR-B, PROGESTERONE RECEPTOR, Progesterone receptor A	progesterone receptor	208305_at
PLD1	AA536939, C85393, Pc-Plc, Phospholipase D1, PLD1A, PLD1B, Plda, Pldb	phospholipase D1, phosphatidylcholine-specific	215723_s_at
PML	1200009E24Rik, AI661194, MYL, PP8675, Retinoic acid receptor, RGD1562602, RNF71, TRIM19	promyelocytic leukemia	211013_x_at
PVR	3830421F03Rik, CD155, D7ERTD458E, FLJ25946, HVED, mE4, NECL5, NECTIN 2 ALPHA, NECTIN-2, Pe4, PVS, TAA1, TAGE4	poliovirus receptor	212662_at
RARRES1	5430417P09RIK, AI662122, TIG1, Tig1/retinoic acid receptor responder 1	retinoic acid receptor responder (tazarotene induced) 1	206391_at
SCGB1D2	BU101, LIPB, LIPOPHILIN B, LPHB	secretoglobin, family 1D, member 2	206799_at
SEL1L	AW493766, IBD2, KIAA4137, mKIAA4137, PRO1063, SEL1, SEL1-LIKE, SEL1H	sel-1 suppressor of lin-12-like (C. elegans)	202063_s_at
SERPINA5	4933415L04, MGC93420, PAI-3, PCI, Pi5 alpha1, PLANH3, PROCI	serpin peptidase inhibitor, clade A (alpha-1 antiproteinase, antitrypsin), member 5	209443_at
SERPINA6	AI265318, AV104445, CBG, MGC112780	serpin peptidase inhibitor, clade A (alpha-1 antiproteinase, antitrypsin), member 6	206325_at
SLC7A5	4f2 light chain, 4F2LC, CD98, CD98 LIGHT CHAIN, CD98LC, D0H16S474E, D16S469E, E16, E16/TA1, hLAT1, LAT1, MPE16, TA1	solute carrier family 7 (cationic amino acid transporter, y+ system), member 5	201195_s_at
SPAM1 (includes EG:6677)	4933439A12Rik, HYA1, HYAL1, HYAL3, HYAL5, MGC108951, MGC26532, PH-20, SPAG15, SPAM1, TESTICULAR HYALURONIDASE	sperm adhesion molecule 1 (PH-20 hyaluronidase, zona pellucida binding)	210536_s_at
TBC1D9	4933431N12RIK, AI847101, AW490653, C76116, KIAA0882, MDR1, RGD1308221	TBC1 domain family, member 9 (with GRAM domain)	212956_at
TFRC	2610028K12Rik, AI195355, AI426448, AU015758, CD71, E430033M20Rik, Mtvr-1, p90, TFNR, TFR, TFR1, TRANSFERRIN RECEPTOR, TRFR	transferrin receptor (p90, CD71)	208691_at
TRPV6	ABP/ZF, Cac, CAT, CAT-L, CAT1, Crac, ECAC2, HSA277909, LP6728, Otrpc3, ZFAB	transient receptor potential cation channel, subfamily V, member 6	206827_s_at

**Figure 1:**
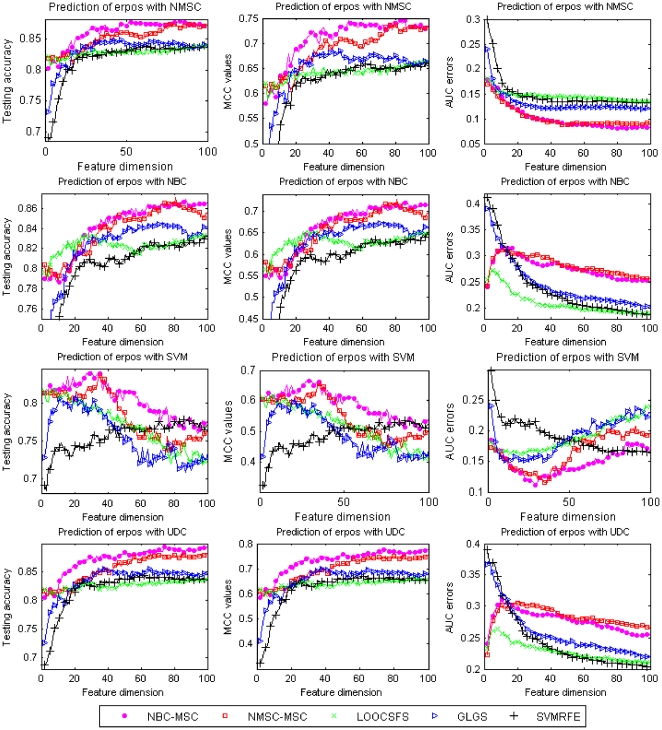
Comparison of different gene selection methods for prediction of erpos status of MAQC-II breast cancer dataset with different learning classifiers. X-axis shows the number of used features and Y-axis shows average values of the testing accuracy (left column), MCC values (middle column), and AUC errors (right column) of twenty-time experiments, respectively.

Figure 1 lists the testing accuracy, MCC values, and AUC errors on prediction of erpos by using two RFA methods: NBC-MSC and NMSC-MSC, as well as GLGS, LOOCSFS, and SVMRFE with the four learning classifiers. Regarding the prediction performance evaluated using testing accuracy and MCC values (left column and middle column, Fig. 1), on average the two RFA methods NBC-MSC and NMSC-MSC outperform other compared gene selection methods. The advantage of RFA by using NMSC and UDC is especially noticeable.

We notice that the performance evaluation using testing accuracy (left column) and MCC (middle column) is consistent, but the performance evaluation using AUC measurement is not always consistent with the evaluation using testing accuracy and MCC. For instance, in applying UDC to the feature sets, RFA methods have the best prediction performance evaluated using testing accuracy and MCC values; however, with respect to performance evaluated using AUC errors RFA is not the best. Regarding the testing performance measured by using AUC errors, the best results have been obtained by using RFA with NMSC classifier. The AUC errors are as low as 0.08, which is much better than the results by using other methods of gene selection.

**Figure 2:**
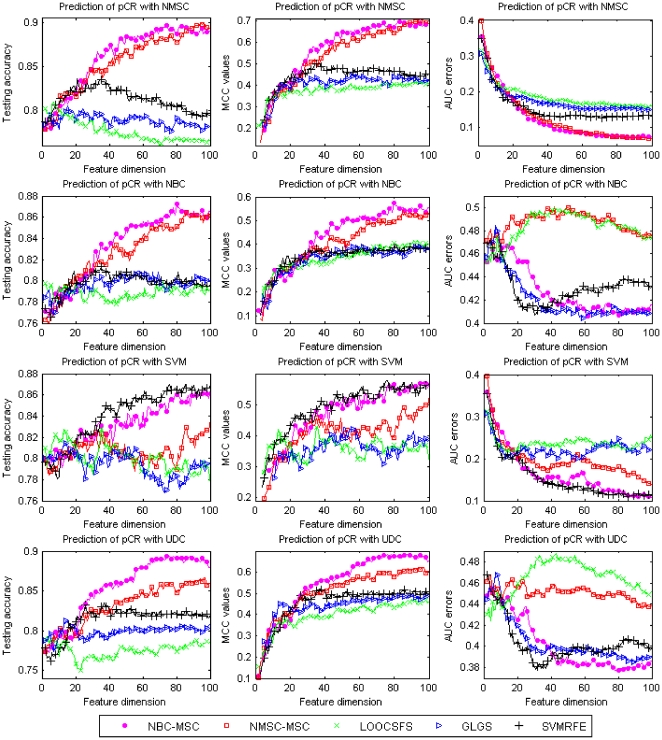
Comparison of different gene selection methods for prediction of pCR status of MAQC-II breast cancer dataset with different learning classifiers. X-axis shows the number of used features and Y-axis shows average values of the testing accuracy (left column), MCC values (middle column), and AUC errors (right column) of twenty-time experiments, respectively.

The prediction results on pCR endpoint, shown in Figure 2, also demonstrate the advantage of RFA methods over other compared methods. All the best prediction results, evaluated by using AUC, MCC and testing accuracy, are obtained by using RFA methods.

**Figure 3:**
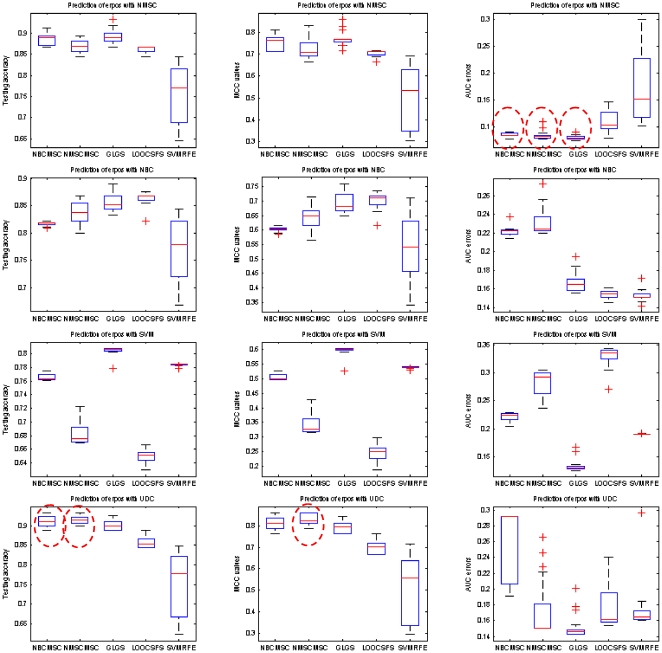
Average erpos prediction performance by using MAQC-II breast cancer dataset with the measurements testing accuracy (left column), MCC values (middle column), and AUC errors (right column), respectively. Classification models are setup based on the best training. In each column, the best combination of gene selection and classifier is highlighted by a red dash circle. If there are multiple best combinations, or the difference of these combinations is not conspicuous, multiple circles are placed.

**Figure 4:**
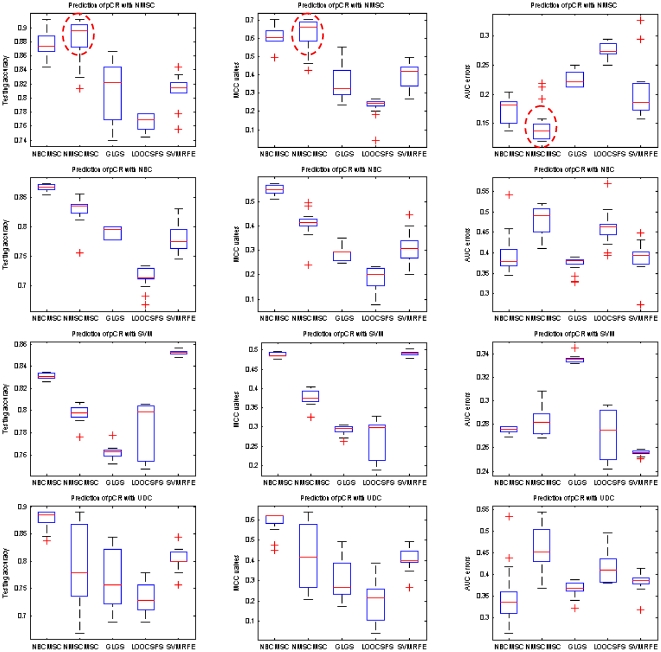
Average pCR prediction performance by using MAQC-II breast cancer dataset with the measurements testing accuracy (left column), MCC values (middle column), and AUC errors (right column), respectively. Classification models are setup based on the best training. In each column, the best combination of gene selection and classifier is highlighted by a dash circle.

Tables 11 and 12 list the average testing under the best training, MS_HR, and the best testing under the best training, HS_HR, for the predictions on erpos and pCR, respectively. Figure 3 and Figure 4 show box-plots of MS_HR values for the predictions on erpos and pCR. The results indicate that the prediction performance depends not only on gene selection but also on learning classifier. In other words, gene selection and classifier are coupled in determining prediction performance. On average, RFA gene selection methods deliver the best performance, followed by GLGS; NMSC classifier outperforms the others with respect to the performance and the consistency across the three measurements. The combination of RFA with NMSC has the best overall prediction performance.

**Table 11:** Mean values and standard errors of HS_HR and MS_HR on ERPOS prediction in the study of MAQC-II breast cancer dataset. In applying each classifier with each measurement, the best prediction value is in bold; the best prediction value of the results using the four learning classifiers is in bold and italic.

MEASURE-MENTS	GENE SELECTION METHOD	MEAN(HS_HR)±STD(HS_HR), %				MEAN(MS_HR)±STD(MS_HR), %			
		NMSC	SVM	NBC	UDC	NMSC	SVM	NBC	UDC
Testing Accuracy	NBC-MSC	89.5±2.2	79.3±2.2	82.9±1.1	91.6±1.7	88.5±1.4	76.5±0.5	81.7±0.3	91.0±1.6
	NMSC-MSC	**90.6±2.8**	73.3±0	85.4±2.8	***93.0±0.8***	86.9±1.5	68.3±1.6	83.7±1.9	***91.6±1.1***
	GLGS	89.7±2.1	**83.8±2.1**	86.8±1.8	90.5±1.4	**89.2±1.7**	**80.3±0.9**	85.7±1.7	90.2±1.3
	LOOCSFS	86.4±0.8	66.7±1.9	**86.9±1.4**	86.3±1.6	86.1±0.8	65.1±0.9	**86.2±1.0**	85.8±1.4
	SVMRFE	77.3±7.2	84.4±0	77.6±6.6	77.5±8.9	75.6±6.7	78.4±0.1	76.7±6.1	75.8±7.6
MCC values	NBC-MSC	76.7±4.5	56.1±4.7	63.1±2.3	81.9±3.7	75.3±3.3	50.5±1.0	60.4±0.7	81.0±3.4
	NMSC-MSC	**79.1±5.8**	44.8±0	66.6±5.6	***84.5±2.4***	72.2±4.1	34.4±3.5	64.4±4.2	***82.7±2.5***
	GLGS	77.4±3.6	**67.1±4.9**	**71.3±3.9**	79.5±3.2	**76.9±3.4**	**59.2±2.3**	**69.7±3.8**	79.1±2.7
	LOOCSFS	70.8±1.7	27.0±5.6	70.9±3.3	69.7±3.3	70.5±1.8	24.4±3.0	70.2±2.9	69.6±3.3
	SVMRFE	51.9±14.5	66.6±0	53.9±11.9	53.8±16.6	51.0±13.9	53.8±0.3	53.5±11.5	52.3±15.0
AUC errors	NBC-MSC	8.5±0.4	19.5±2.2	22.2±0.5	25.7±4.4	8.5±0.3	22.2±0.7	22.2±0.5	25.7±4.4
	NMSC-MSC	8.4±0.7	23.5±0.4	23.1±1.3	17.1±3.6	8.4±0.7	28.2±2.3	23.1±1.3	17.1±3.6
	GLGS	***8.0±0.4***	**10.3±1.8**	16.7±1.0	**15.1±1.5**	***8.0±0.4***	**13.4±1.1**	16.7±1.0	**15.1±1.5**
	LOOCSFS	11.1±2.2	32.3±2.7	15.5±0.5	17.5±2.6	11.1±2.2	32.9±1.8	15.5±0.5	17.5±2.6
	SVMRFE	17.3±6.1	14.1±0	**15.3±0.6**	17.3±2.9	17.3±6.1	19.1±0.1	**15.3±0.6**	17.3±2.9

**Table 12:** Mean values and standard errors of HS_HR and MS_HR on pCR prediction in the study of MAQC-II breast cancer dataset. In applying each classifier with each measurement, the best prediction value is in bold; the best prediction value is in bold and italic of the results by using four classifiers.

MEASURE-MENTS	GENE SELECTION METHOD	MEAN(HS_HR)±STD(HS_HR), %				MEAN(MS_HR)±STD(MS_HR), %			
		NMSC	SVM	NBC	UDC	NMSC	SVM	NBC	UDC
Testing Accuracy	NBC-MSC	88.7±2.3	**88.9±0**	***90.9±1.0***	**88.6±0.8**	87.5±1.9	83.1±0.3	**86.6±0.6**	**87.7±1.6**
	NMSC-MSC	**90.4±2.1**	86.7±0.0	85.3±2.2	79.6±7.7	***88.6±2.7***	79.8±0.7	82.9±2.0	78.9±7.4
	GLGS	81.2±4.1	81.9±0.8	79.4±1.0	77.0±5.2	80.8±4.3	76.2±0.6	79.1±1.0	76.5±5.2
	LOOCSFS	77.0±1.1	82.1±0.5	72.1±1.7	74.1±3.1	76.6±1.2	78.1±2.5	71.4±1.8	73.2±2.7
	SVMRFE	81.9±2.9	**88.9±0**	79.1±2.6	80.7±1.9	80.9±2.7	**85.3±0.2**	77.9±2.3	80.3±1.8
MCC values	NBC-MSC	62.5±6.7	**63.9±0**	***69.7±3.4***	**60.7±2.8**	61.3±5.9	48.8±0.6	**54.7±2.1**	**59.2±5.0**
	NMSC-MSC	**68.4±6.5**	55.2±0	47.7±7.4	43.9±16.0	***63.3±7.9***	37.8±1.9	40.9±5.0	42.2±14.9
	GLGS	36.2±9.5	43.0±3.5	29.3±3.5	30.3±10.2	35.4±9.8	29.2±1.2	28.4±2.8	30.0±10.3
	LOOCSFS	23.4±4.9	38.5±3.5	18.7±4.7	21.6±11.0	23.0±5.1	26.3±5.1	18.2±5.0	21.1±10.7
	SVMRFE	40.9±7.9	62.5±0.0	32.6±6.9	41.1±4.7	39.4±7.4	**49.1±0.7**	30.5±5.5	40.7±4.9
AUC errors	NBC-MSC	17.2±2.2	23.2±1.1	40.0±5.6	**34.8±6.3**	17.2±2.2	27.5±0.3	40.0±5.6	**34.8±6.3**
	NMSC-MSC	***14.4±3.0***	19.1±0	47.9±3.7	46.0±5.1	***14.4±3.0***	28.2±1.1	47.9±3.7	46.0±5.1
	GLGS	22.6±1.3	29.3±0	**37.4±1.8**	36.7±1.8	22.6±1.3	33.5±0.3	**37.4±1.8**	36.7±1.8
	LOOCSFS	27.5±1.3	22.8±1.4	45.8±3.8	41.6±3.1	27.5±1.3	27.1±2.2	45.8±3.8	41.6±3.1
	SVMRFE	20.0±4.4	**18.8±5.7**	38.7±3.4	38.4±2.0	20.0±4.3	**25.6±0.2**	38.7±3.4	38.4±2.0

### Results on MAQC-II Multiple Myeloma Dataset

**Figure 5:**
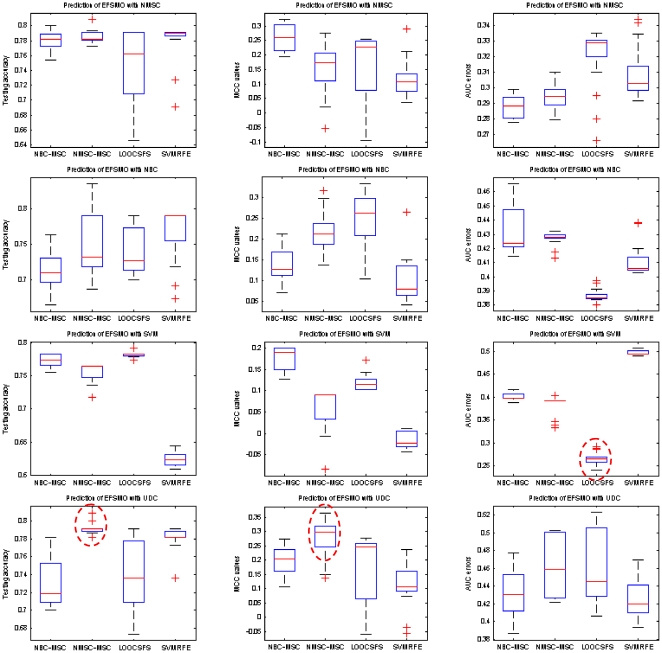
Average EFSMO prediction performance by using MAQC-II multiple myeloma dataset with the measurements testing accuracy (left column), MCC values (middle column), and AUC errors (right column), respectively. Classification models are setup based on the best training. In each column, the best combination of gene selection and classifier is highlighted by a dash circle.

**Figure 6:**
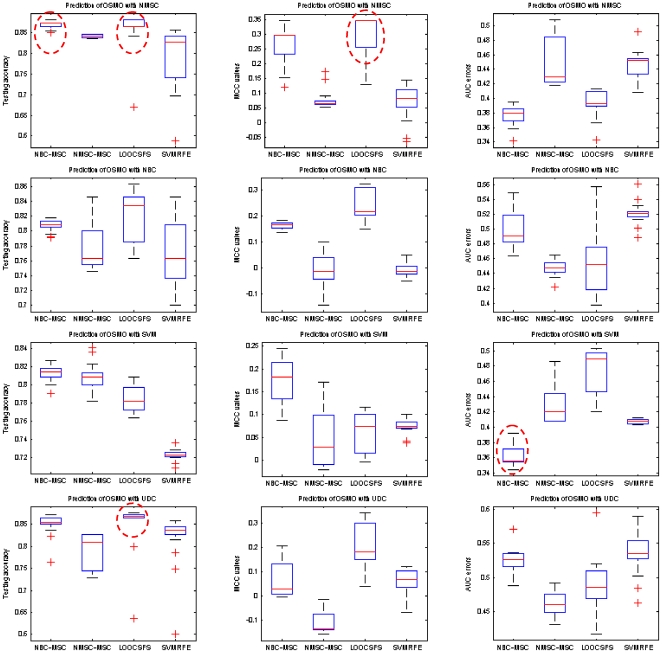
Average OSMO prediction performance by using MAQC-II multiple myeloma dataset with the measurements testing accuracy (left column), MCC values (middle column), and AUC errors (right column), respectively. Classification models are setup based on the best training. In each column, the best combination of gene selection and classifier is highlighted by a dash circle. If there are multiple best combinations, or the difference of these combinations is not conspicuous, multiple circles are placed.

Figures 5 and 6 show the box-plots of average testing values for EFSMO and OSMO, the classification models are based on the best training. We did not apply GLGS because it would take an excessive amount of time for the identification of the feature sets on the multiple myeloma dataset. Experimental results again manifest that gene selection is strictly coupled to learning classifier in performance measurement. On average, RFA methods and LOOCSFS are superior to SVMRFE.

## Discussion

Due to a huge number of variables and small sample size, there are complicated interactions and relations among genes as well as high redundancy information with microarray data. The selection of predictive models that depend on selected features and employed classifiers is extremely important for the classification of microarray data and for the further biological function analysis/validation. Machine learning and data mining techniques provide us with a powerful approach to the study of the relationship among genes. Based on supervised learning and similarity measurements, we propose a Recursive Feature Addition (RFA), recursively employ supervised learning to obtain the highest training accuracy and add a subsequent gene based on the similarity between the chosen features and the candidates to minimize the redundancy within the feature set. We believe this RFA method captures more informative and differently expressed genes than other methods. Experimental comparisons are performed by using two MAQC-II microarray datasets, breast cancer and multiple myeloma. Our studies show that the method of gene selection is strictly paired with learning classifier, which determines the final predictive model by using training data. In other words, the best classification models under different learning classifiers are associated with different methods of gene selection. Using several popular learning classifiers including NMSC, NBC, SVM, and UDC, on average, the best method of gene selection is RFA, followed by GLGS, LOOCSFS, and SVMRFE. Regarding compared learning classifiers, NMSC outperforms the others with respect to testing performance, stabilization, and consistency.

Biological function analysis based on MAQC-II breast cancer dataset finds that our applied feature selection methods including RFA, GLGS, LOOCSFS, and SVMRFE can generate features containing a significant portion of known cancer related genes for both pCR and erpos endpoints (Tables 1–10). Although the cancer related gene number is not absolutely correlated with the prediction performance of various methods of feature selection, the remarkable cancer related genes in the features indicate that the feature selection methods including RFA, GLGS, LOOCSFS, and SVMRFE could produce biologically meaningful features, which will convince the users to apply them for phenotype prediction.

**Figure 7:**
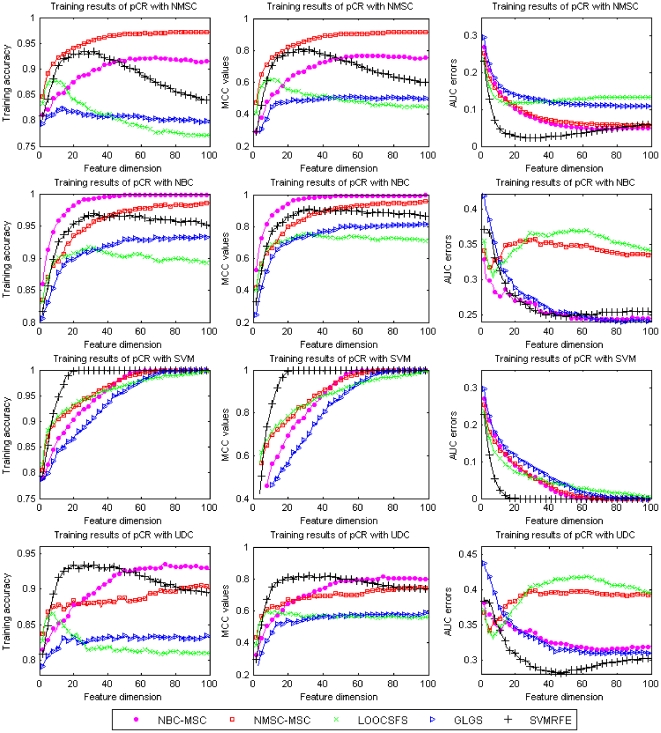
Comparison of different gene selection methods for the training of pCR endpoint of MAQC-II breast cancer dataset using the four classifiers. X-axis shows the number of used features and Y-axis shows average values of the training accuracy (left column), MCC values (middle column), and AUC errors (right column) of twenty-time experiments, respectively.

In all results of the five gene selection methods with the four learning classifiers, on average, the combination of gene selections of NMSC-MSC and NBC-MSC with the learning classifier of NMSC has performed the best, regarding the comprehensive evaluation criteria of testing accuracy, MCC values, and AUC errors. It should be noted that the gene selection methods of NMSC-MSC and NBC-MSC are not always the best over the four learning classifiers, in other words, the best models among different learning classifiers are associated with different gene selection methods. The selection of the best model with the use of a specific learning classifier is normally based on the training and the evaluation criteria. Under an evaluation criterion with the use of some learning classifier, the best training model among the five gene selection methods is selected as the best model under the learning classifier. To select the best model among the four learning classifiers, the best models among the four learning classifiers are compared and the model obtaining the highest evaluation score is generally selected the best among the five gene selection methods and the four learning classifiers. For instance, Figure 7 demonstrates the average training performance of MAQC-II breast cancer dataset over twenty times, with the measurements of training accuracy, MCC values, and AUC errors, for the classification of pCR endpoint status. Regarding the comprehensive evaluation of the three criteria, the best classification models are obtained by using gene selection method NMSC-MSC for learning classifier NMSC, NBC-MSC for NBC classifier, and NBC-MSC and SVMRFE for UDC classifier. With the use of SVM classifier, although the gene selection method of SVMRFE first hits the best training as the feature dimension increases, almost all gene selection methods achieve the best before the feature number increases to 100, in such case, it is hard for us to determine the best model with the use of learning classifier SVM. In the limit of feature number, we can say that SVMRFE is the best for SVM classifier. The comparison between the training (Figure 7) and the testing (Figure 2) shows that such selection of the best model among the various methods of gene selection and various learning classifiers is reasonable.

Regarding different evaluation criteria, the gene selection associated with the best model under a learning classifier may be different, as shown in Figure 7; with the use of learning classifier NMSC, the best model is obtained by using gene selection method of NMSC-MSC, evaluated by training accuracy and MCC values, the best model is associated with SVMRFE, evaluated by AUC errors. Overall, NMSC-MSC is the best for learning classifier NMSC. Figure 7 shows that dozens of the best training models are obtained by using different methods of gene selection with the use of SVM classifier. One possible solution for the selection of the best model under SVM classifier is to divide all data points into training set, validation set, and testing set. The training set is used to construct training models, the validation set is used to select the best model by applying the validation data to the best training models and selecting the best training model that produce the best validation result. The testing set is used for prediction or testing. The selection of the best model using SVM classifier is very interesting and challenging, especially in the case of small data points and huge number of features. Although the topic is beyond the scope of this paper, it is worthy to be explored in the future study.

## Materials and Methods

### MAQC-II Breast Cancer Dataset

The breast cancer dataset used in the MAQC-II project is used to predict the pre-operative treatment response (pCR) and estrogen receptor status (erpos) [28]. The normalization was provided by MAQC-II project using standard procedure (i.e., MAS 5.0 for Affymetrix platform). It was originally grouped into two groups: a training set containing 130 samples (33 positives and 97 negatives for pCR, 80 positives and 50 negatives for erpos), and a validation set containing 100 samples (15 positives and 85 negatives for pCR, 61 positives and 39 negatives for erpos).

### MAQC-II Multiple Myeloma Dataset

We take the MAQC-II multiple myeloma dataset to predict the overall survival milestone outcome (OSMO, 730-day cutoff) and to predict event-free survival milestone outcome (EFSMO, 730-day cutoff). For OSMO label information, there are 51 positives and 289 negatives in original training set, 27 positives and 187 negatives in original validation set; as for EFSMO label information, there are 84 positives and 256 negatives in original training set, 34 positives and 180 negatives in original validation set [28]. The normalization was provided by MAQC-II project research group.

### Feature Selection

#### Supervised recursive learning

Our method of recursive feature addition (RFA) employs supervised learning to achieve the best training accuracy and uses statistical similarity measures to choose the next variable with the least dependence on, or correlation to, the already identified variables as follows:

1. Insignificant genes are removed according to their statistical insignificance. Specifically, a gene with a high p-value is usually not differently expressed and therefore has little contribution to classification of microarray data. To reduce the computational load, those insignificant genes are removed.
2. Each individual gene is selected by supervised learning. A gene with highest classification accuracy is chosen as the most important feature or the first element of the feature set. If multiple genes achieve the same highest classification accuracy, the one with the lowest *p*-value measured by test-statistics (e.g., score test), is identified as the first element. At this point the chosen feature set, **G**_1_, contains just one element, *g*_1_, corresponding to the feature dimension one.
3. The (N*+*1)^st^ dimensional feature set, **G**_N__+1_ = {*g*_1_, *g*_2_, …, *g*_N_, *g*_N__+1_} is obtained by adding *g*_N__+1_ to the N*^th^* dimensional feature set, **G**_N_ = {*g*_1_, *g*_2_, …, *g*_N_}. The choice of *g*_N+1_ is processed as follows:
4. Add each gene *g_i_* (*g_i_* 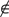 **G**_N_) into **G**_N_ and have the classification accuracy of the feature set **G**_N_ 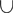 {*g_i_*}. The *g_i_* (*g_i_* 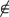 **G**_N_) associated with the group, **G**_N_ 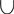 {*g_i_*} that obtains the highest classification accuracy, is the candidate for *g*_N+1_ (not yet *g*_N+1_). Considering the large number of variables, it is very likely that multiple features correspond to the same highest classification accuracy, these multiple candidates are placed into the set **C**, but only one candidate in **C** will be identified as *g*_N+1_. How to make the selection is described next.

#### Candidate feature addition

To find a most informative (or least redundant) candidate for *g*_N+__1_, we measure the statistical similarity between the chosen features and each candidate. We design a similarity measurement with the use of a widely-used Pearson's correlation coefficient [30].

Suppose *g_n_* (*g_n_* 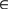 **G**_N_, *n* = 1, 2, …, N) is a chosen gene, *g_c_* (*g_c_* 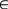 **C**) is a candidate gene, and cor stands for the function of Pearson's correlation coefficient. The sum of the square of the correlation SC that is calculated as follows:
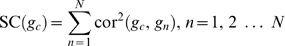
(1)


Then selection of *g*_N+1_ is based on Minimal value of the Square of the Correlation (MSC), that is,
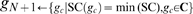
(2)


In the methods mentioned above, a feature is recursively added to the chosen feature set based on supervised learning and the similarity measurement. In our experiments we choose naïve bayes classier (NBC) and nearest means scale classifier (NMSC) [29] for supervised learning, NBC and NMSC-based RFA feature selection methods are denoted as NBC-MSC and NMSC-MSC, respectively.

### Model Implementation and Comparison

Cross-validation is a technique for estimating how accurately a predictive model will perform in practice. Generally, the data are partitioned into complementary subsets, one subset (called the training set) is used for constructing the predictive model and the other subset (called the validation set or testing set) is used for validation. To reduce variability, multiple rounds are performed using different partitions, and the validation results are averaged over all rounds. There are three common types of cross-validation:

1. Repeated random sub-sampling validation. This technique randomly splits the dataset intro training and testing data. The results are then averaged over the splits. The advantage over K-fold cross validation (described below) is that the portion of the training/testing split is not dependent on the number of iterations (folds);
2. K-fold cross-validation. The original sample is partitioned into K subsamples. A single subsample is retained as the validation data for testing the model and the remaining K-1 subsamples are used as training data. The cross-validation process is repeated K times with each of the K subsamples used exactly once as the validation data;
3. Leave-one-out cross-validation. It uses a single observation from the original sample as the validation data and the remaining observations as the training data. It is the same as a K-fold cross validation with K being equal to the number of observations in the original sample. Leave-one-out cross-validation is often computationally expensive.

Considering the high computational requirement of leave-one-out cross-validation and the insufficiency of one time K-fold cross-validation, we took the strategy of repeated random sub-sampling validation. In the model implementation, we mixed all the training data points and validation points. In each experiment, we randomly chose 80% of samples for training and the remaining 20% of samples for testing. Twenty experiments were performed (this strategy is approximately equal to performing 5-fold cross validation four times). The average testing performances, evaluated in terms of testing accuracy, MCC values, and AUC errors, were compared. The learning classifiers UDC, NBC, NMSC, and SVM were employed for training and testing.
